# Long noncoding RNA LYPLAL1-AS1 regulates adipogenic differentiation of human mesenchymal stem cells by targeting desmoplakin and inhibiting the Wnt/β-catenin pathway

**DOI:** 10.1038/s41420-021-00500-5

**Published:** 2021-05-15

**Authors:** Yanlei Yang, Junfen Fan, Haoying Xu, Linyuan Fan, Luchan Deng, Jing Li, Di Li, Hongling Li, Fengchun Zhang, Robert Chunhua Zhao

**Affiliations:** 1 Institute of Basic Medical Sciences Chinese Academy of Medical Sciences, School of Basic Medicine Peking Union Medical College, Peking Union Medical College Hospital, Center of Excellence in Tissue Engineering Chinese Academy of Medical Sciences, Beijing Key Laboratory (No. BZO381), 100005 Beijing, China; 2 Department of Rheumatology and Clinical Immunology, Peking Union Medical College Hospital, Clinical Immunology Center, Chinese Academy of Medical Sciences and Peking Union Medical College, The Ministry of Education Key Laboratory, 100005 Beijing, China

**Keywords:** Adult stem cells, Cell biology

## Abstract

Long noncoding RNAs are crucial factors for modulating adipogenic differentiation, but only a few have been identified in humans. In the current study, we identified a previously unknown human long noncoding RNA, LYPLAL1-antisense RNA1 (LYPLAL1-AS1), which was dramatically upregulated during the adipogenic differentiation of human adipose-derived mesenchymal stem cells (hAMSCs). Based on 5′ and 3′ rapid amplification of cDNA ends assays, full-length LYPLAL1-AS1 was 523 nt. Knockdown of LYPLAL1-AS1 decreased the adipogenic differentiation of hAMSCs, whereas overexpression of LYPLAL1-AS1 enhanced this process. Desmoplakin (DSP) was identified as a direct target of LYPLAL1-AS1. Knockdown of DSP enhanced adipogenic differentiation and rescued the LYPLAL1-AS1 depletion-induced defect in adipogenic differentiation of hAMSCs. Further experiments showed that LYPLAL1-AS1 modulated DSP protein stability possibly via proteasome degradation, and the Wnt/β-catenin pathway was inhibited during adipogenic differentiation regulated by the LYPLAL1-AS1/DSP complex. Together, our work provides a new mechanism by which long noncoding RNA regulates adipogenic differentiation of human MSCs and suggests that LYPLAL1-AS1 may serve as a novel therapeutic target for preventing and combating diseases related to abnormal adipogenesis, such as obesity.

## Introduction

Abnormal adipogenic differentiation is closely related to obesity, although the regulatory mechanisms involved remain to be completely elucidated. Mesenchymal stem cells (MSCs) are multipotent stem cells and are considered to be the main source of adipocytes^1^. Many studies have demonstrated their potential use in treating obesity and obesity-related diseases^2–4^. Therefore, elucidating the regulatory mechanism of adipogenesis in MSCs is of great importance for developing novel targets and therapies for treating abnormal adipogenic-related diseases. Adipogenesis from MSCs is mediated by a series of complex processes, including the commitment of MSCs to preadipocytes that then differentiate into mature adipocytes^1^. The mechanism regulating this process involves the Wnt/β-catenin pathway, Hedgehog pathway, transforming growth factor-β superfamily, and insulin-like growth factor signaling pathways as key mediators^1^. Many transcription factors such as peroxisome proliferator-activated receptor-γ (PPARγ) and members of the CCAAT/enhancer-binding proteins (C/EBPs) also regulate this process^5^. However, the full scope of transcription factors and pathways involved in MSC adipogenesis remains to be well defined.

Long noncoding RNAs (LncRNAs) represent a subfamily of transcripts that are >200 nt and have limited protein-encoding potential^6,7^. They have pivotal roles in various biological processes, such as X chromosome inactivation^8^, chromatin remodeling^9–11^, transcriptional repression^12^, cancer metastasis^13^, and stem cell fate determination^14–18^. For example, lncRNA PGC1β-OT1 reciprocally regulates mouse MSC adipogenic and osteogenic differentiation through antagonizing miR-148a-3p and enhancing the KDM6B effect^19^. LncRNA GAS5 serves as a sponge for miR-18a to inhibit the adipogenic differentiation of human MSCs^20^. LncRNA ADINR regulates adipogenesis of human MSCs by directly binding to PA1 and then modulates histone modification of the C/EBPα locus, thereby activating the transcription of C/EBPα^21^. To date, only a few lncRNAs have been identified that regulate the adipogenic differentiation of human MSCs. Several lncRNAs have been reported to regulate adipogenic differentiation, lipid metabolism, and obesity^22–24^, and given their tissue-specific expression patterns, lncRNAs can serve as biomarkers for obesity^25^.

In the current study, we identified a previously unknown human lncRNA, LYPLAL1-antisense RNA1 (LYPLAL1-AS1), and showed that it has a role in adipogenic differentiation. Our findings provide new insights into understanding the mechanism of adipogenic differentiation of hAMSCs and suggest that LYPLAL1-AS1 may serve as a novel therapeutic target for adipogenesis-related disorders.

## Results

### Identification, validation, and characterization of LYPLAL1-AS1 during the adipogenic differentiation of hAMSCs

**Fig. 1:**
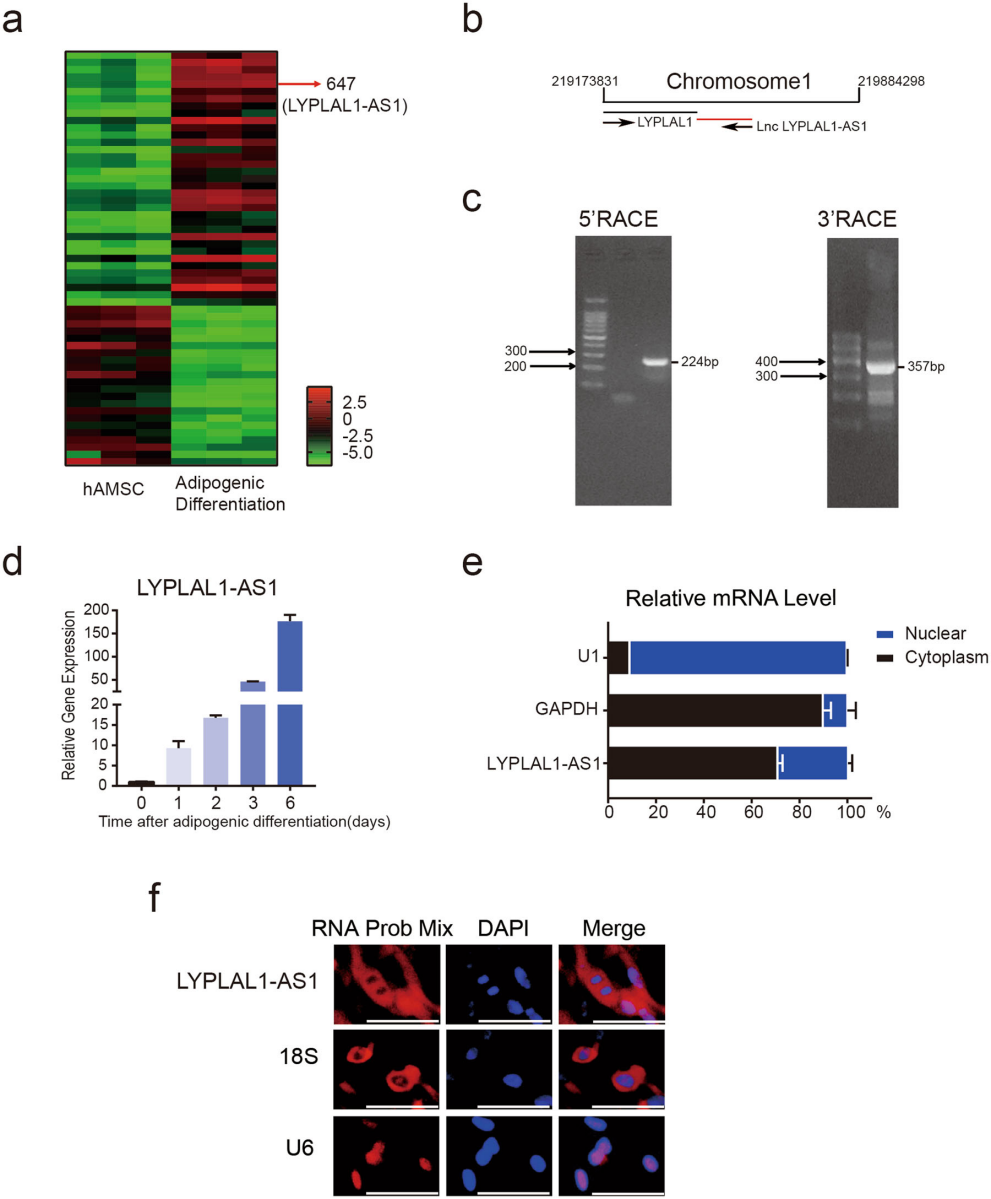
Identification, validation, and characterization of LYPLAL1-AS1 during the adipogenic differentiation of hAMSCs. **a** Hierarchical clustering of significantly differentially expressed lncRNAs on day 3 after adipogenic differentiation as compared with matched undifferentiated hAMSCs from three donors. The red arrow indicates LYPLAL1-AS1. **b** Location of lncRNA LYPLAL1-AS1 on chromosome 1. **c** Rapid amplification of cDNA ends (RACE) for the 5′ end (left) and 3′ end (right) was performed to determine the full length of LYPLAL1-AS1. **d** qRT-PCR analysis verified the LYPLAL1-AS1 expression profile at the indicated time points after hAMSC adipogenic differentiation. **e** Fractionation of LYPLAL1-AS1 in hAMSCs followed by qRT-PCR. *GAPDH* mRNA served as a cytoplasmic control. *U1* mRNA served as a nuclear control. **f** RNA fluorescence in situ hybridization (FISH) assay for LYPLAL1-AS1 in hAMSCs. 18S was the cytoplasmic mRNA control. U6 was the nuclear RNA control. The quantitative data were normalized to *GAPDH*, *n* = 3; data are shown as the mean ± SD; scale bars: 50 µm.

hAMSCs isolated from human adipose tissue are fibroblast-like, plastic-adherent cells that have the potential to differentiate into adipocytes and osteocytes; they are positive for CD29, CD73, CD44, CD90, and CD105 expression and are negative for CD206, CD34, HLA-DR, CD45, and CD106 expression (Fig. S1a–c)^26^. To systematically identify functional lncRNAs that are involved in the regulation of hAMSC adipogenic differentiation, we performed microarray analysis before and after adipogenic differentiation of hAMSCs from three donors. Next, lncRNA expression profiles were analyzed according to stringent criteria (fold change ≥ 2, expression value ≥ 3, and *P* < 0.05). We identified 57 lncRNAs that were differentially expressed (35 were upregulated and 22 were downregulated) among hAMSCs and adipocytes (Fig. 1a). Among the top upregulated lncRNAs, we noticed an uncharacterized and not well-conserved lncRNA, LYPLAL1-AS1, which is located downstream of LYPLAL1 on chromosome 1 and is transcribed from the opposite direction relative to LYPLAL1. This lncRNA was dramatically upregulated during adipogenic differentiation of hAMSCs (Fig. 1a, b and Fig. S1d, e), which was also confirmed by a real-time quantitative reverse transcription-PCR (qRT-PCR) assay (Fig. 1d). 5′ and 3′ Rapid amplification of cDNA ends (RACE) assays identified that the full length of LYPLAL1-AS1 was 523 nt (Fig. 1c and Fig. S1f).

The specific subcellular location of lncRNAs is essential for understanding their functions and mechanisms. Cell fractionation followed by qRT-PCR demonstrated that LYPLAL1-AS1 displayed mainly a cytoplasmic distribution (>70%) (Fig. 1e). As controls, glyceraldehyde 3-phosphate dehydrogenase (GAPDH) displayed a cytoplasmic distribution, whereas U1 displayed a nuclear distribution (Fig. 1e). The RNA fluorescent in situ hybridization (FISH) assay further validated that LYPLAL1-AS1 was distributed mainly in the cytoplasm. 18S rRNA and U6 were used as controls (Fig. 1f).

### LYPLAL1-AS1 depletion inhibits adipogenic differentiation of hAMSCs, whereas its overexpression promotes this process

**Fig. 2:**
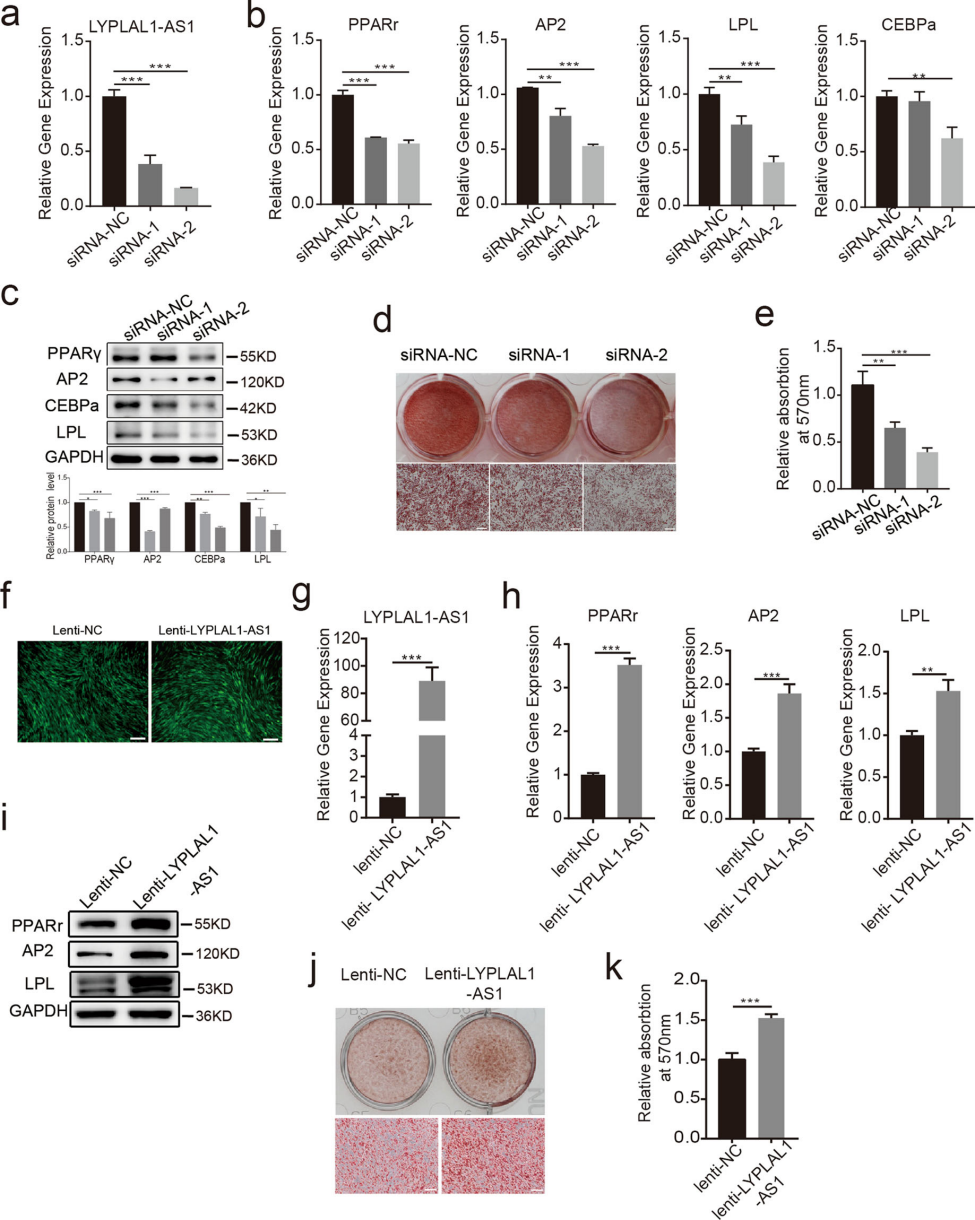
LYPLAL1-AS1 depletion inhibits adipogenic differentiation of hAMSCs, whereas its overexpression promotes this process. **a** LYPLAL1-AS1 was silenced in hAMSCs using two independent siRNAs (siRNA-1 and siRNA-2). The knockdown efficiency was verified by qRT-PCR as compared with the negative control (si-NC). **b**. qRT-PCR analysis of adipogenic markers (PPARγ, AP2, LPL, and CEBPα) in hAMSCs with LYPLAL1-AS1 knockdown and in control hAMSCs on day 3 after adipogenic induction. **c** Western blot analysis of adipogenic markers (PPARγ, AP2, LPL, and CEBPα) in LYPLAL1-AS1 knockdown hAMSCs or control hAMSCs on days 3 and 6 after adipogenic induction. **d**, **e** Oil red O staining of adipose lipids in hAMSCs treated with LYPLAL1-AS1 siRNAs and in control hAMSCs on day 10 after adipogenic induction (**d**) and quantification of the oil red O staining (**e**). **f**, **g** hAMSCs were transduced with lentivirus overexpressing LYPLAL1-AS1 (Lenti-LYPLAL1-AS1) or control lentivirus (Lenti-NC), and ectopic overexpression efficiency was detected by fluorescence observation (**f**) and qRT-PCR (**g**). **h**, **i** qRT-PCR (**h**) and western blot (**i**) analysis of adipogenic markers (PPARγ, AP2, and LPL) in hAMSCs that overexpressed LYPLAL1-AS1 and in control hAMSCs on day 3 after adipogenic induction. **j**, **k** Oil red O staining of adipose lipids in hAMSCs that overexpressed LYPLAL1-AS1 and in control hAMSCs on day 10 after adipogenic induction (**j**) and quantification of the oil red O staining (**k**). GAPDH was used as internal controls for western blotting. The quantitative data were normalized to GAPDH, *n* = 3; data are shown as the mean ± SD; **P < 0.01, ***P < 0.001; scale bars: 200 µm.

To evaluate the effect of LYPLAL1-AS1 on adipogenic differentiation, we silenced LYPLAL1-AS1 in hAMSCs using two pairs of specific short interfering RNAs (siRNAs) (Fig. 2a). Then, hAMSCs were induced to differentiate into adipocytes. qRT-PCR and western blot analyses showed that LYPLAL1-AS1 knockdown resulted in notably decreased expression of adipogenic markers PPARγ, AP2, LPL, and CEBPα at both messenger RNA (mRNA) and protein levels, as compared with cells transfected with the control siRNA (Fig. 2b, c). Lipid droplets in adipocytes are visualized with the reagent oil red O, which can be used to determine the efficiency of adipogenic differentiation^27^. Consistently, knockdown of LYPLAL1-AS1 decreased the adipogenic differentiation efficiency of hAMSCs (Fig. 2d, e). To exclude any off-target effects of the siRNAs used for LYPLAL1-AS1 knockdown, we performed further knockdown assays using RNase-H-based antisense oligonucleotides (ASOs). Likewise, ASO targeting of LYPLAL1-AS1 not only downregulated the levels of adipogenic markers PPARγ, AP2, and LPL (Fig. S2a–c) but also decreased the adipogenic differentiation efficiency of hAMSCs (Fig. S2d, e).

To further confirm the function of LYPLAL1-AS1 in adipogenic differentiation of hAMSCs, we stably overexpressed full-length LYPLAL1-AS1 using lentivirus in hAMSCs (Fig. 2f, g). As verified by qRT-PCR, ectopic overexpression of LYPLAL1-AS1 significantly promoted the expression of adipogenic markers PPARγ, AP2, and LPL at the mRNA level (Fig. 2h). The western blot assay revealed that overexpression of LYPLAL1-AS1 (Lenti-LYPLAL1-AS1) notably promoted adipogenic differentiation as compared with hAMSCs infected with empty vector control (Lenti-NC), as indicated by decreased protein levels of the adipogenic markers PPARγ, AP2, and LPL (Fig. 2i). In addition, LYPLAL1-AS1 overexpression increased the adipogenic differentiation efficiency of hAMSCs, as indicated by oil red O staining (Fig. 2j, k). Collectively, these data indicated that LYPLAL1-AS1 played a positive role in the adipogenic differentiation of hAMSCs.

### LYPLAL1-AS1 is positively correlated with adipogenic markers in human adipose tissues and promotes de novo adipogenesis in vivo

**Fig. 3:**
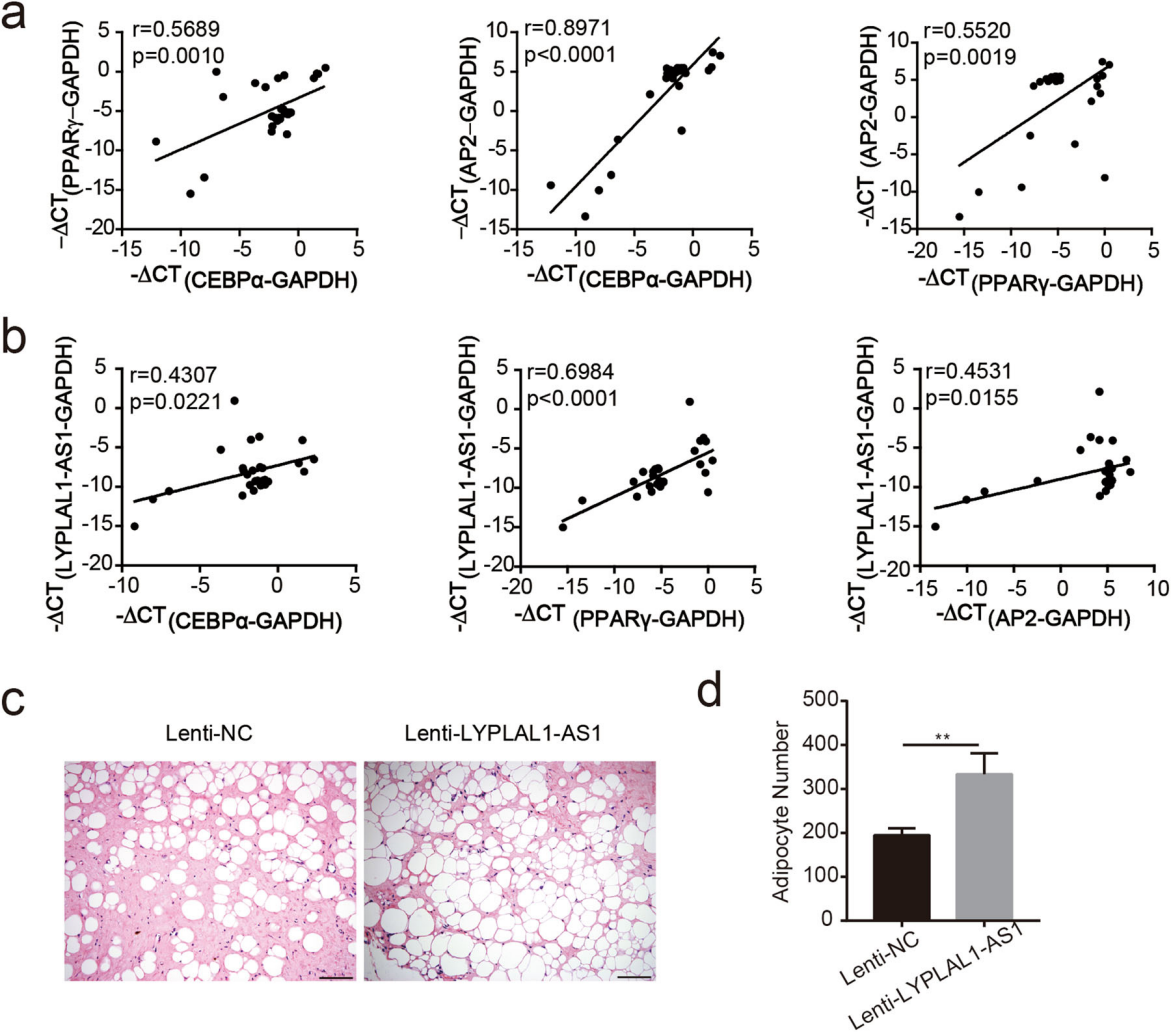
LYPLAL1-AS1 is positively correlated with adipogenic markers in human adipose tissues and promotes de novo adipogenesis in vivo. **a** qRT-PCR analysis of adipogenic markers (PPARγ, AP2, and CEBPα) and the correlation analysis for each marker pair among 28 clinical adipose tissue samples. The internal control was GAPDH. **b** qRT-PCR analysis of LYPLAL1-AS1 and adipogenic markers (PPARγ, AP2, and CEBPα) among 28 clinical adipose tissue samples. **c**, **d** Representative H&E staining images for de novo adipogenesis in NOD/SCID nude mice injected with the indicated hAMSCs (**c**) and quantification of the number of adipocytes 8 weeks after injection (**d**). The quantitative data were normalized to GAPDH, *n* = 3; data are shown as the mean ± SD, *n* = 28; **P < 0.01; scale bars: 200 µm.

Adipogenic differentiation is driven by adipogenic genes, e.g., PPARγ and CEBPα^5,28,29^, which serve as transcriptional factors for downstream genes involved in adipogenesis. Although adipogenesis is not necessarily related to obesity, it serves an important role in the development of obesity^30,31^. To further confirm the significance of LYPLAL1-AS1 on adipogenic differentiation, we verified the correlation of LYPLAL1-AS1 expression with adipogenic markers in human adipose tissue. When we examined 28 human adult adipose samples, we noted that gene expression profiles for adipogenic markers PPARγ, AP2, and CEBPα were positively correlated with each other (Fig. 3a) and that LYPLAL1-AS1 expression was positively correlated with PPARγ, AP2, and CEBPα expression individually (Fig. 3b), suggesting that LYPLAL1-AS1 may exert a positive effect on the adipogenic differentiation of hAMSCs and may serve as a novel target for the prevention of adipogenic-related disorders.

To assess the potential effect of LYPLAL1-AS1 on adipogenesis in vivo, MSCs transfected with specific lentivirus (Lenti-NC and Lenti-LYPLAL1-AS1) were subjected to adipogenic differentiation medium for 3 days and were then mixed with Matrigel and injected subcutaneously into nonobese diabetic/severe-combined immunodeficiency (NOD/SCID) nude mice (Fig. S3a). These adipose plugs were harvested 8 weeks later and subjected to hematoxylin and eosin (H&E) staining. There was a remarkable increase in the number of adipose cells in the plugs from mice injected with Lenti-LYPLAL1-AS1 hAMSCs in contrast to those injected with Lenti-NC hAMSCs (Fig. 3c, d). To exclude the possibility that the adipose cells observed in the plugs originated from the mice, sections taken from the adipose plugs were immunohistochemically stained for expression of GFP (as encoded by the lentivirus vector) to label specifically the injected hAMSCs (Fig. S3b).

### Identification of endogenous protein partners associated with LYPLAL1-AS1 in hAMSCs

**Fig. 4:**
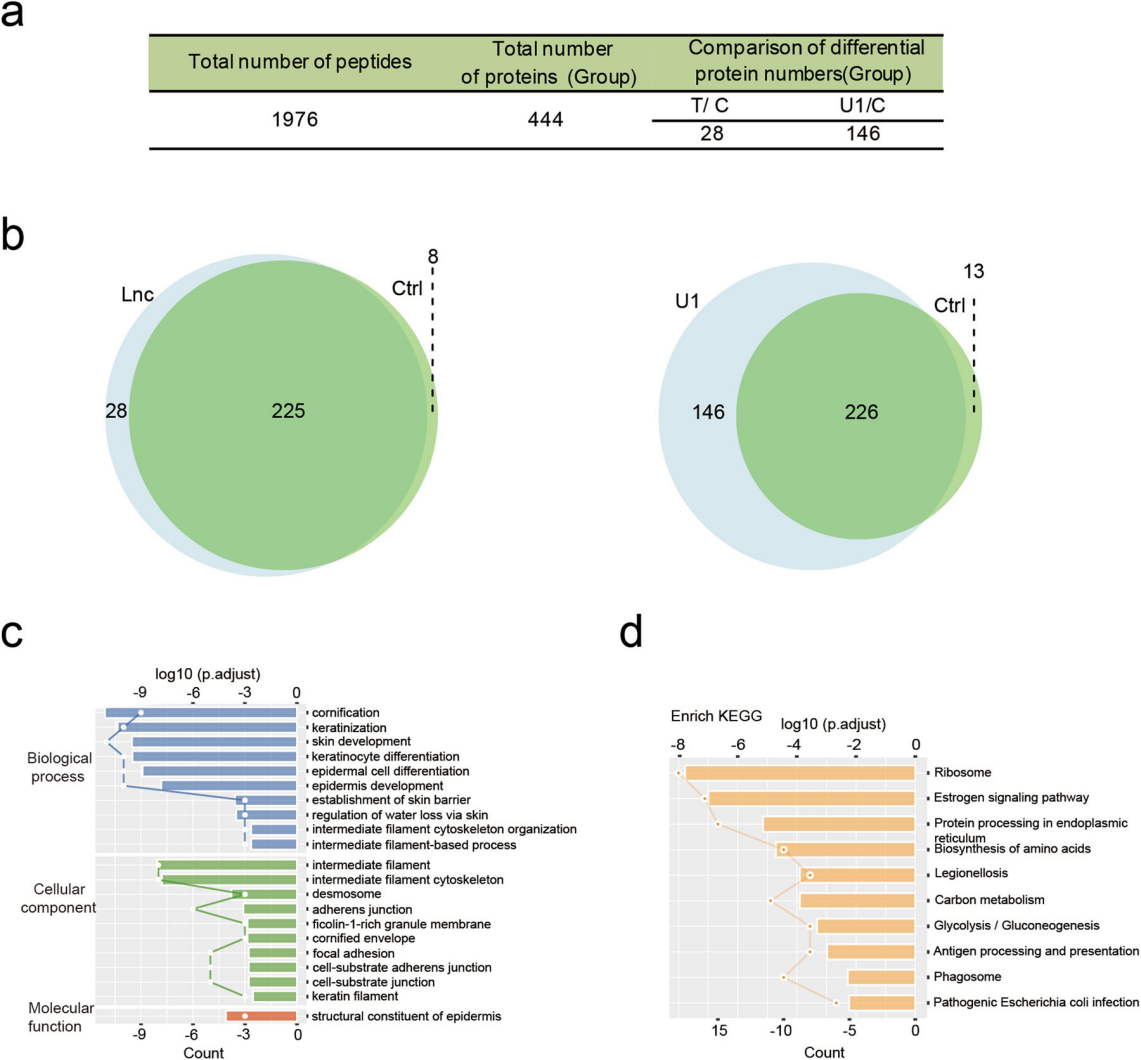
Identification of endogenous protein partners associated with LYPLAL1-AS1 in hAMSCs. **a** Results from the ChIRP-MS assay. The experimental group (T, Test group, LYPLAL1-AS1 group), the negative control group (C), and the positive control group (U1) were individually analyzed quantitatively. **b** ChIRP-MS identified proteins that bind LYPLAL1-AS1 (left) and U1 (right); light blue indicates proteins that bind LYPLAL1-AS1 and U1, and light green indicates proteins that bind in the negative control group. **c**, **d** GO analysis (**c**) and KEGG analysis (**d**) of the identified proteins that directly bind to LYPLAL1-AS1. The *y*-axis shows the GO terms and the KEGG pathway names. The top *x*-axis shows the significance of the enrichment as log 10 (*P* value).

To investigate the mechanism by which LYPLAL1-AS1 regulates the adipogenic differentiation of hAMSCs, we performed a comprehensive identification of RNA-binding proteins by mass spectrometry (ChIRP-MS) assay, which is an ideal strategy for capturing in vivo lncRNA–protein interactions with high yield without genetic tagging^32^. The workflow of the ChIRP-MS procedure is shown in Fig. S4a. We first designed a set of five specific probes labeled with biotin to pull down the proteins that directly bind to LYPLAL1-AS1 (Fig. S4b). Over 40% of LYPLAL1-AS1 was selectively pulled down without enrichment of the housekeeping control GAPDH or actin mRNAs (Fig. S4c). To ensure the reliability of the results, a positive control was added to the experiment in the form of a U1 small nuclear RNA-specific probe. Finally, the experimental LYPLAL1-AS1 group (Test), the negative control group (C), and the positive control group (U1) were included in the experiment (Fig. S4d). Subsequent analysis was based on the quantitative information shown in Fig. 4a. In total, we identified 28 specific LYPLAL1-AS1-binding proteins (Fig. 4b, left) and 146 specific U1-binding proteins (Fig. 4b, right) from this assay.

A Gene Ontogeny (GO) enrichment analysis revealed that the direct LYPLAL1-AS1-binding proteins were enriched for biological processes such as epidermal cell differentiation and cellular component terms such as cell-substrate adherens junction and desmosome (Fig. 4c). Kyoto Encyclopedia of Genes and Genomes pathway annotations revealed that the specific LYPLAL1-AS1-binding proteins were involved in carbon metabolism and the estrogen signaling pathway (Fig. 4d), both of which are involved in regulating the homeostasis or differentiation of adipose tissues^33–35^. In addition, protein interaction analysis of the specific binding proteins using STRINGdb revealed a significant enrichment in interactions among the 28 proteins (Fig. S4e).

### Desmoplakin negatively regulates the adipogenic differentiation of hAMSCs

**Fig. 5:**
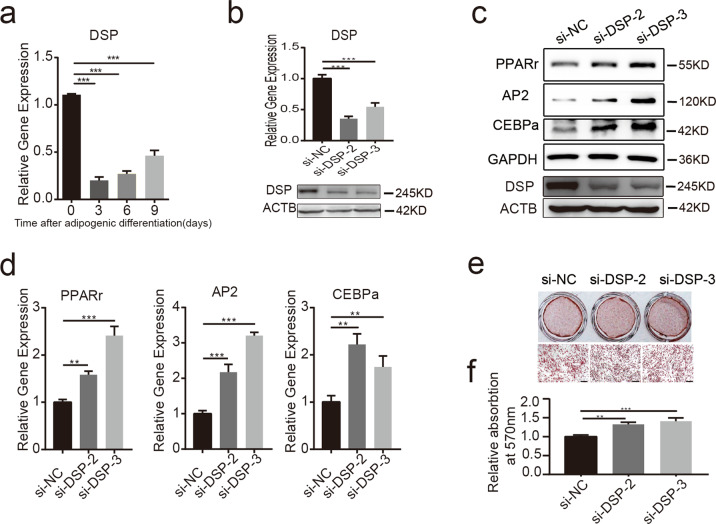
Desmoplakin negatively regulates the adipogenic differentiation of hAMSCs. **a** qRT-PCR analysis verified the DSP expression profile at the indicated time points after adipogenic differentiation of hAMSCs. **b** DSP was silenced in hAMSCs using two independent siRNAs (si-DSP-2 and si-DSP-3). The knockdown efficiency was verified by qRT-PCR (upper) and western blotting (lower) as compared with the negative control (si-NC). **c**, **d** Western blot analysis (**c**) and qRT-PCR analysis (**d**) of adipogenic markers (PPARγ, AP2, and CEBPα) in DSP knockdown hAMSCs and control hAMSCs on day 3 after adipogenic induction. **e**, **f** Oil red O staining of adipose lipids in DSP knockdown hAMSCs and control hAMSCs on day 10 after adipogenic induction (**e**) and quantification of the oil red O staining (**f**). GAPDH and ACTB were used as internal controls for western blotting. The quantitative data were normalized to GAPDH, *n* = 3; data are shown as the mean ± SD; **P < 0.01, ***P < 0.001; scale bars: 200 µm.

Among the 28 identified proteins that directly bind LYPLAL1-AS1, we focused on desmoplakin (DSP). Although DSP negatively regulates adipocyte differentiation of cardiac fibro-adipocyte progenitors^36^, its function in the adipogenic differentiation of hAMSCs has not yet been studied. As verified by qRT-PCR, DSP was downregulated during the adipogenic differentiation of hAMSCs (Fig. 5a). We then determined whether DSP had a role in regulating this process. We first silenced the expression of DSP using two siRNAs against DSP in hAMSCs (Fig. 5b). Both qRT-PCR and western blot analyses showed that DSP depletion resulted in an elevation of the adipogenic markers PPARγ, AP2, and CEBPα after adipogenic induction as compared with control cells (Fig. 5c, d). Moreover, DSP depletion significantly increased the adipogenic differentiation efficiency of hAMSCs as verified by oil red O staining (Fig. 5e, f). Collectively, these data indicated that DSP played a negative role in the adipogenic differentiation of hAMSCs, which was opposite to that of LYPLAL1-AS1.

### LYPLAL1-AS1 regulates adipogenic differentiation of hAMSCs potentially by modulating DSP protein stability

**Fig. 6:**
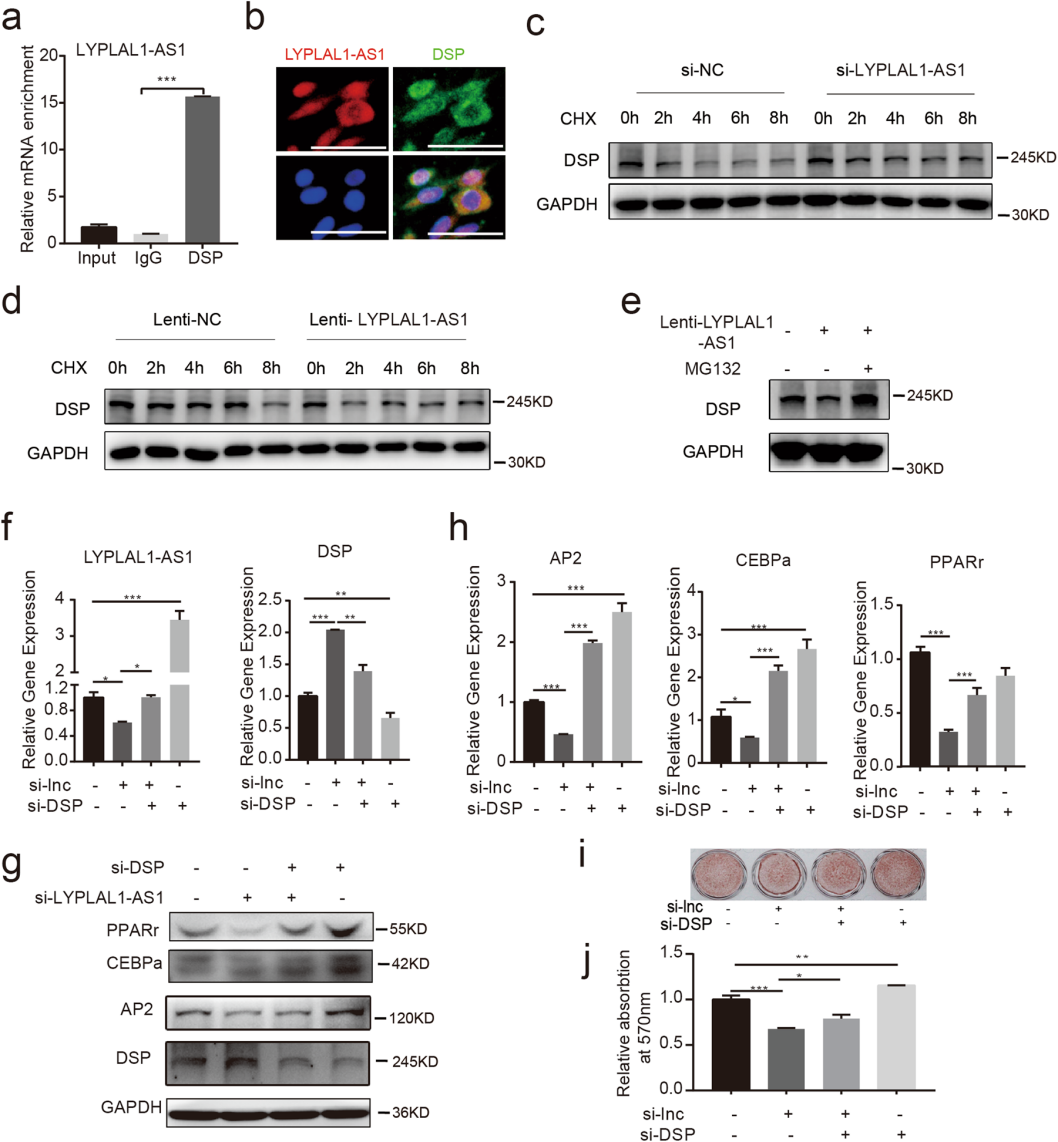
LYPLAL1-AS1 regulates adipogenic differentiation of hAMSCs potentially by modulating DSP protein stability. **a** RIP assay to assess the interaction between DSP and LYPLAL1-AS1 in hAMSCs. Antibodies against DSP (DSP) were used to immunoprecipitate lncRNAs, followed by qRT-PCR with LYPLALA-AS1-specific primers. IgG was used as the negative control. Relative abundances were compared to 1% of the input. **b** FISH assay of LYPLAL1-AS1 transcripts followed by immunofluorescence assay for DSP showed the colocalization of LYPLAL1-AS1 transcripts and DSP in the cytoplasm. **c**, **d** Western blot analysis of DSP stability in the presence of LYPLAL1-AS1 knockdown (**c**) and LYPLAL1-AS1 overexpression (**d**) in hAMSCs and with treatment with 5 µg/ml cycloheximide (CHX) for 0, 2, 4, 6, and 8 h. **e** Western blot analysis of DSP in hAMSCs that overexpress LYPLAL1-AS1 and in the absence and presence of MG132 (10 μM) treatment for 4 h. **f** LYPLAL1-AS1 knockdown and control hAMSCs were transfected with siRNAs targeting DSP. The levels of LYPLAL1-AS1 and DSP were detected by qRT-PCR. **g**, **h** Western blotting (**g**) and qRT-PCR (**h**) assays detected adipogenic marker genes (PPARγ, AP2, and CEBPα) in hAMSCs after LYPLAL1-AS1 knockdown, DSP knockdown, and LYPLAL1-AS1 knockdown followed by DSP knockdown. **i**, **j** Oil red O staining assay (**i**) and subsequent quantification (**j**) in hAMSCs after LYPLAL1-AS1 knockdown, DSP knockdown, and LYPLAL1-AS1 knockdown followed by DSP knockdown. GAPDH was used as the internal control for the western blot assays. The quantitative data were normalized to GAPDH, *n* = 3; data are shown as the mean ± SD; *P < 0.05, **P < 0.01, ***P < 0.001; scale bars: 50 µm.

To further verify the interaction between DSP and LYPLAL1-AS1, we performed an RNA immunoprecipitation (RIP) assay to immunoprecipitate endogenous lncRNAs using the DSP antibody. As we expected, LYPLAL1-AS1 was indeed detected in the immunoprecipitated complex (Fig. 6a). Moreover, FISH of LYPLAL1-AS1 followed by immunofluorescence staining for DSP showed that they were colocalized in the cytoplasm (Fig. 6b). As LYPLAL1-AS1 displayed a mainly cytoplasmic distribution, we speculated whether LYPLAL1-AS1 could modulate DSP protein decay. To evaluate the effect of LYPLAL1-AS1 on DSP protein stability, hAMSCs transduced with Lenti-LYPLAL1-AS1 or control vector (Lenti-NC) were treated with cycloheximide (CHX; 5 μg/ml), which inhibits protein synthesis, for 0, 2, 4, 6, and 8 h. The half-life of DSP was ~2 h based on our previous results (Fig. S5a). As detected by western blotting, knockdown of LYPLAL1-AS1 resulted in delayed DSP degradation (Fig. 6c). In contrast, DSP protein degradation was accelerated when LYPLAL1-AS1 was overexpressed (Fig. 6d). We next used MG132 to block the function of the proteasome to inhibit the degradation of proteins^37^. DSP protein levels accumulated significantly when treated with MG132 for 4 h based on our previous results (Fig. S5b). As verified by western blotting, the level of DSP decreased when LYPLAL1-AS1 was overexpressed in the absence of MG132, whereas the level of DSP accumulated significantly when proteasome function was inhibited by MG132 (Fig. 6e). Collectively, these results suggested that LYPLAL1-AS1 overexpression decreased DSP levels possibly by promoting DSP degradation via the proteasome.

We next confirmed whether LYPLAL1-AS1 regulated adipogenic differentiation of hAMSCs in a DSP-dependent manner. We carried out a rescue assay to verify whether DSP silencing could rescue the LYPLAL1-AS1 knockdown-induced defect in adipogenic differentiation of hAMSCs. We silenced DSP in LYPLAL1-AS1 knockdown hAMSCs or control cells (Fig. 6f). As mentioned above, LYPLAL1-AS1 knockdown resulted in lower expression of adipogenic markers PPARγ, AP2, and CEBPα, whereas DSP silencing significantly increased adipogenic marker expression. The inhibiting effect of LYPLAL1-AS1 knockdown on adipogenic differentiation of hAMSCs was reversed when DSP was subsequently silenced, as indicated by detecting the expression of key adipogenic markers PPARγ, AP2, and CEBPα with western blotting (Fig. 6g), qRT-PCR assays (Fig. 6h), and oil red O staining for lipid droplet generation (Fig. 6i, j). These data demonstrated that LYPLAL1-AS1 regulates adipogenic differentiation of hAMSCs in a DSP-dependent manner.

Collectively, these findings indicated that DSP, the protein stability of which was potentially regulated by LYPLAL1-AS1, was responsible for the positive effect of LYPLAL1-AS1 on adipogenic differentiation of hAMSCs.

### Wnt/β-catenin pathway is involved in LYPLAL1-AS1/DSP-regulated adipogenic differentiation of hAMSCs

**Fig. 7:**
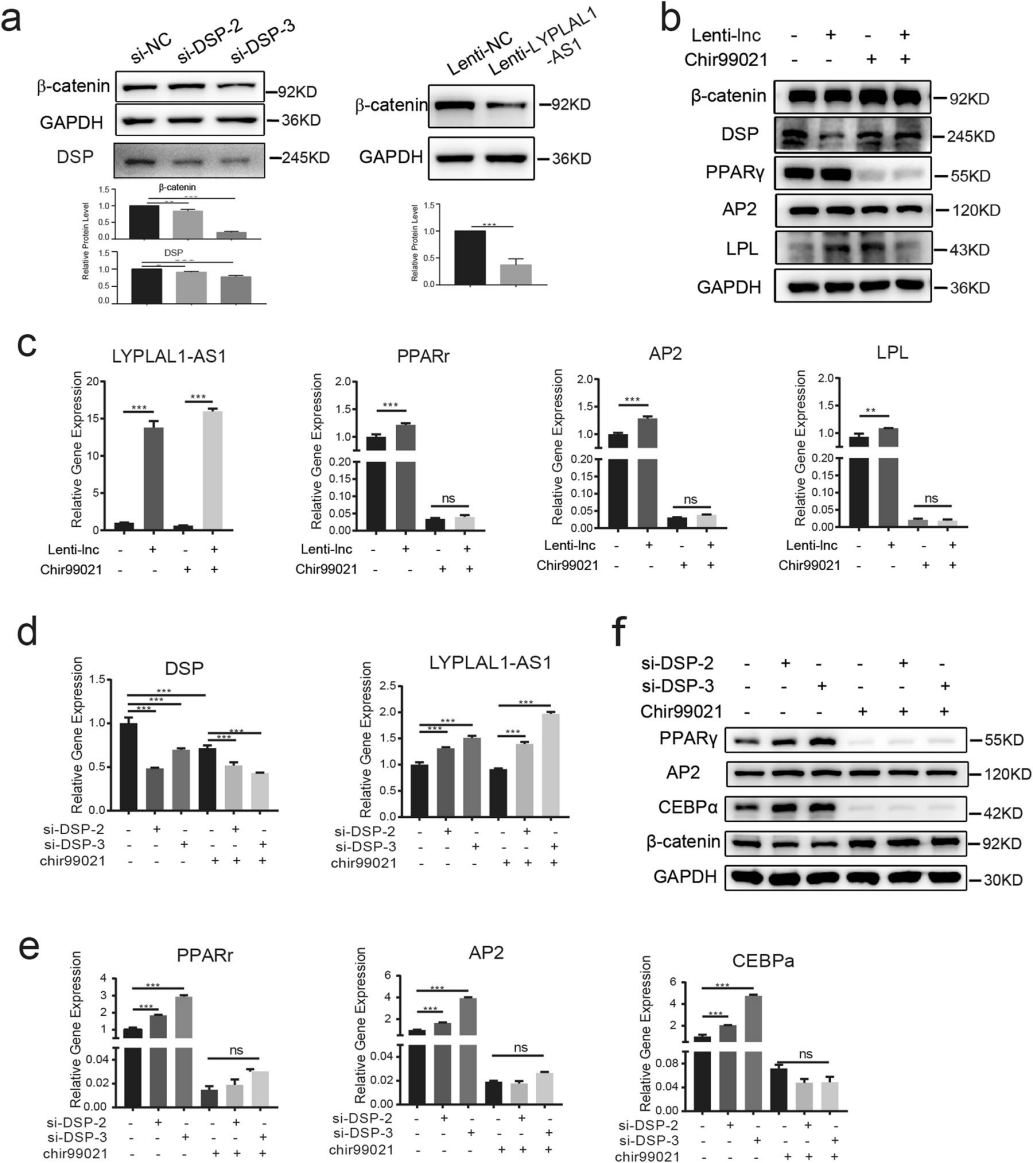
Wnt/β-catenin pathway is involved in LYPLAL1-AS1/DSP-regulated adipogenic differentiation of hAMSCs. **a** Western blot analysis of β-catenin and DSP in DSP knockdown hAMSCs (si-DSP-2, si-DSP-3) and control hAMSCs (si-NC) and western blot analysis of β-catenin in hAMSCs that overexpress LYPLAL1-AS1 (Lenti-LYPLAL1-AS1) and control hAMSCs (Lenti-NC). **b** Western blot analysis of β-catenin, DSP, and adipogenic markers (PPARγ, AP2, and LPL) in hAMSCs that overexpress LYPLAL1-AS1 (Lenti-LYPLAL1-AS1; Lenti-lnc+) and control hAMSCs (Lenti-NC; Lenti-lnc−) after treatment without or with chir99021. **c** qRT-PCR analysis of LYPLAL1-AS1 and adipogenic markers (PPARγ, AP2, and LPL) in hAMSCs that overexpress LYPLAL1-AS1 and control hAMSCs after treatment without or with chir99021. **d** qRT-PCR analysis of LYPLAL1-AS1 and DSP in DSP knockdown (si-DSP-2, si-DSP-3) and control hAMSCs after treatment without or with chir99021. **e** qRT-PCR analysis of adipogenic markers (PPARγ, AP2, and CEBPα) in DSP knockdown and control hAMSCs after treatment without or with chir99021. **f** Western blot analysis of β-catenin and adipogenic markers (PPARγ, AP2, and CEBPα) in DSP knockdown and control hAMSCs after treatment without or with chir99021.GAPDH was used as the internal control for the western blot assays. The quantitative data were normalized to GAPDH, *n* = 3; data are shown as the mean ± SD; *P < 0.05, **P < 0.01, ***P < 0.001; n.s., not significant.

Next, we examined the pathway involved in LYPLAL1-AS1/DSP-regulated adipogenic differentiation of hAMSCs. Based on our previous results, LYPLAL1-AS1 overexpression or DSP knockdown resulted in the downregulation of β-catenin protein (Fig. 7a), which indicated that the Wnt/β-catenin pathway may be involved. To evaluate the effects of the Wnt/β-catenin pathway on LYPLAL1-AS1/DSP-regulated adipogenic differentiation, hAMSCs infected with Lenti-LYPLAL1-AS1 or with control vector Lenti-NC were treated with chir99021 (3 μM), an activator of the Wnt/β-catenin pathway^38^, for 24 h. As verified by qRT-PCR and western blot assays, the expression levels of adipogenic markers increased when LYPLAL1-AS1 was overexpressed, whereas this effect was blocked by the presence of chir99021 (Fig. 7b, c). Similarly, the ability of DSP depletion to promote adipogenic differentiation in hAMSCs was also blocked by chir99021 (Fig. 7d–f). Collectively, these data revealed that Wnt/β-catenin pathway inhibition was necessary for LYPLAL1-AS1/DSP-regulated adipogenic differentiation of hAMSCs.

## Discussion

Several studies have reported the function of lncRNAs in regulating adipogenic differentiation;^19–21,39,40^ however, only a few of these lncRNAs have been identified in humans. Most lncRNAs are poorly conserved among different species, and therefore studies of lncRNAs that have specific regulatory effects on human adipogenic differentiation are more likely to be of clinical significance. The specific cellular localization of a lncRNA dictates its biological function and mechnism^41,42^. Nuclear lncRNAs usually form complexes with RNA-binding proteins to carry out their functions in regulating transcriptional processes and modulating subcellular structures such as chromosome scaffolding, chromatin remodeling, alternative splicing, and epigenetic control of transcription^43–45^, all of which have been studied extensively. The cytoplasmic lncRNAs are comparatively less well understood but can also form complexes with RNA-binding proteins to affect cytoplasmic events at the post-transcriptional level, such as regulating protein localization and turnover, mRNA translation, and stability^46,47^. Herein, we identified a previously unknown human lncRNA, LYPLAL1-AS1, that had a mainly cytoplasmic distribution, suggesting that it may perform its function at the post-transcriptional level.

Understanding the specific proteins that interact with individual lncRNAs is crucial to understanding the biological functions of those lncRNAs. DSP is mainly expressed in cardiac myocytes, where it is a component of intercalated disks. Misregulation of DSP is the main cause of arrhythmogenic cardiomyopathy^48,49^, as reported, depletion of DSP results in the promotion of adipocyte differentiation among cardiac fibro-adipocyte progenitors^36^. Similarly, DSP played a negative role in the adipogenic differentiation of hAMSCs, which was opposite to the function of LYPLAL1-AS1. The detailed mechanism by which DSP affects the adipogenic differentiation of hAMSCs awaits further study.

Interestingly, we observed the upregulation of LYPLAL1-AS1 at the mRNA level when DSP was silenced. As the majority of LYPLAL1-AS1 was present in the cytoplasm (>70%), we speculate that LYPLAL1-AS1 might be a downstream effector of DSP at the post-transcriptional level. Thus, to evaluate the effect of DSP on LYPLAL1-AS1 mRNA stability, hAMSCs transfected with si-NC or si-DSP were treated with actinomycin D (5 μg/ml), which inhibits RNA polymerase and blocks transcription^17^. Knockdown of DSP improved the RNA stability of LYPLAL1-AS1 (Fig. S5c). Hence, we hypothesize that a regulatory feedback mechanism exists between LYPLAL1-AS1 and DSP, although the specific regulatory feedback of LYPLAL1-AS1 and DSP awaits further investigation. In addition, besides the protein stability, the DSP mRNA level was also modulated by LYPLAL1-AS1 (Fig. 6f, right). We, therefore, performed the ChIRP-sequencing assay using LYPLAL1-AS1 probes to uncover the putative regulatory genomic DNA-binding locations of LYPLAL1-AS1. Unfortunately, no noticeable binding signal was identified for DSP, which suggested that LYPLAL1-AS1 did not regulate DSP expression by directly binding to the DSP promoter or another genomic region. Moreover, at the post-translational level, DSP mRNA stability was not influenced by LYPLAL1-AS1 knockdown as showed in Fig. S5d. Therefore, another mechanism could exist concerning the regulation of LYPLAL1-AS1 at the DSP mRNA level, and further studies are required to address this problem. Thus, in the present study, we provide one possible mechanism by which LYPLAL1-AS1 regulates adipogenic differentiation of hAMSCs potentially by modulating DSP protein stability.

Cytoplasmic lncRNAs can influence protein expression programs by controlling protein degradation pathways through the ubiquitin or proteasome machinery^47^. For example, Yang and colleagues found that lncRNA-p21 is transcriptionally activated by the hypoxia-induced protein (HIF-1α), and its transcripts then bind to the von Hippel-Lindau protein, thereby preventing HIF-1α ubiquitination degradation^50^. We found that LYPLAL1-AS1 colocalized with DSP in the cytoplasm and was able to promote DSP protein degradation possibly via the proteasome; however, the specific mechanism needs to be further studied. Our findings help to advance our understanding of the mechanisms by which cytoplasmic lncRNA regulates its target proteins.

lncRNA sequences are poorly conserved across different species^51^, which we found to be the case for LYPLAL1-AS1. This makes it difficult for us to generate conditional knockout mice to study the effects of LYPLAL1-AS1 on adipogenesis in vivo. Based on the results from our study and other studies^52,53^, in vivo subcutaneous Matrigel plugs are a comparatively feasible method for testing the de novo adipogenesis ability of a lncRNA.

Here, we show for the first time that the lncRNA LYPLAL1-AS1 positively regulates adipogenic differentiation of hAMSCs by directly targeting DSP, potentially modulating DSP protein stability, and inhibiting the Wnt/β-catenin pathway. LncRNAs have specific expression patterns, unique sequences, and secondary structures and thus are ideal targets for small molecules or other types of drugs^54^. Further identification of functional lncRNAs, as well as screening for unknown lncRNA-interacting proteins during MSC adipogenic differentiation, will help to develop new strategies for the prevention and treatment of diseases related to abnormal adipogenic differentiation, such as obesity.

## Materials and methods

### Preparation of clinical adipose specimens and isolation, culture, and differentiation of hAMSCs

hAMSCs and hAMSC-derived RNA were obtained from human adipose tissue from patients undergoing liposuction according to procedures approved by the Ethics Committee of the Chinese Academy of Medical Sciences and School of Basic Medicine Peking Union Medical College.

Human adipose samples were each incubated with TRIzol reagent (Invitrogen, Waltham, MA, USA) for total RNA extraction. The isolated RNA was stored at −80 °C for subsequent analysis of lncRNA and adipogenic-associated gene expression (the specific RNA extraction and quantitative reverse transcription-PCR (qRT-PCR) procedure were described below.)

hAMSCs were isolated from human adipose tissue and cultured according to our previous studies^55–57^. Briefly, adipose tissue was washed twice with D-Hanks’ buffer containing penicillin–streptomycin and centrifuged at 800 × *g* for 3 min. The wash solution was discarded, and 0.2% collagenase P (Sigma-Aldrich, USA) was added to the adipose tissue at a ratio of 3:1 adipose volume/collagenase P solution, and the samples were digested at 37 °C on a shaking table for 20–30 min until no obvious lumps were remaining. The digested product was diluted in D-Hanks’ buffer and filtered through a 100-μm sieve to remove any undigested tissue. Additional D-Hanks’ buffer was added to the adipose tissue filtrate to a final volume of 50 ml, and the solution was centrifuged at 800 × *g* for 10 min. This step was repeated two more times to wash away the blood cells and collagenase P. Cells were then cultured in T75 flasks (Thermo Fisher Scientific, USA) in Dulbecco’s modified Eagle’s medium (DMEM)/F12 (Corning, USA) containing 2% fetal bovine serum (FBS; Gibco, Grand Island, NY, USA), 10 ng/ml platelet-derived growth factor bb (Sigma-Aldrich), 10 ng/ml epidermal growth factor (Sigma-Aldrich), 10^–4^ M ascorbic acid 2-phosphate (Sigma-Aldrich), 10^–8^ M dexamethasone (Sigma-Aldrich), 10^–9^ M insulin transferrin selenium (Gibco), and 100 U/ml penicillin and 100 μg/ml streptomycin (both from Gibco), in a humidified incubator at 37 °C with 5% CO_2_. The culture medium was replaced every 2–3 days, and cells were passaged at 90% confluency. Cells at passages 3−5 were used in the following experiments.

For adipogenic differentiation, hAMSCs at passage 3 were exposed to adipogenic differentiation medium, consisting of high glucose-DMEM (Gibco) supplemented with 10% FBS (Gibco), 1 µM dexamethasone (Sigma-Aldrich), 0.5 mM isobutylmethylxanthine (Sigma-Aldrich), and 1 mM ascorbic acid (Sigma-Aldrich) as described^21^. Adipogenic differentiation efficiency was determined by oil red O (Sigma-Aldrich) staining.

hAMSCs were induced to undergo osteogenic differentiation in H-DMEM supplemented with 10% FBS (Gibco, Carlsbad, CA, USA), 10 mM β-glycerophosphate (Sigma-Aldrich, St. Louis, MO, USA), 0.5 mM l-ascorbic acid (Sigma-Aldrich), and 0.01 mM dexamethasone (Sigma-Aldrich) as described^27^.

### Flow cytometry

hAMSCs at passage 3 were harvested and washed twice using D-Hanks’ buffer and then were incubated with phycoerythrin-conjugated antibodies against CD29, CD73, CD44, CD90, CD105, CD206, CD34, CD45, CD106, and HLA-DR (BD Biosciences) or isotype control antibodies (BD Biosciences) for 20 min at 4 °C in the dark. Cells were then washed twice and filtered through a 400-mesh sieve before being analyzed on an Accuri C6 flow cytometer (BD Biosciences) with CFlow Plus software (BD Biosciences).

### Microarray analysis

To identify adipogenic differentiation-related lncRNAs, hAMSCs were induced to undergo adipogenic differentiation as described above, and total RNA was isolated using TRIzol reagent (Invitrogen, USA) on days 0 and 9. hAMSCs from three donors were analyzed in this study. Sample processing and hybridization were conducted by Cnkingbio Biotechnology (Beijing, China) with Affymetrix mRNA and lncRNA microarray chips. To find differentially expressed lncRNAs, we compared lncRNA expression from each sample before versus after adipogenic differentiation (day 0 versus day 9) with the following cut-off criteria: fold change ≥ 2, expression value ≥ 3, and *P* value < 0.05.

### Oil red O staining

Adipogenic differentiation cells at 8–10 days in culture were washed twice with D-Hanks’ solution and fixed with 4% paraformaldehyde for 10 min at room temperature. Then, the cells were washed three times with D-Hanks’ solution and stained with filtered oil red O solution (Sigma-Aldrich, USA) (stock solution: 3 mg/ml in isopropanol; working solution: 60% oil red O stock solution and 40% distilled water) for 30 min at 37 °C. After staining, the cells were washed under flowing water for 1 min and then visualized and imaged by light microscopy. To quantify the dying cells, the oil red O staining was extracted by isopropanol, and the OD value was measured at 570 nm by a microplate reader.

### Alkaline phosphatase (ALP) and Alizarin red staining

ALP staining was performed using the ALP Staining Kit (Institute of Hematology and Blood Diseases Hospital, Chinese Academy of Medical Sciences, Tianjin, China). Alizarin red staining was performed to detect matrix mineralization deposition during the later stage of osteogenesis as described^27^. In brief, cells in culture were washed twice with phosphate-buffered saline (PBS), fixed in 4% paraformaldehyde for 10 min, and then washed with distilled water and stained with 1% Alizarin red (Leagene, Beijing, China) for 30 min at room temperature. Then, cells were washed with distilled water to remove the unbound dye, and matrix calcification was visualized as red deposits under light microscopy.

### RNA isolation and qRT-PCR

Total RNA was extracted from cultured cells or fresh adipose tissue by TRIzol reagent according to the manufacturer’s instructions. Then, reverse transcription of 2 µg RNA from each sample was performed using M-MLV Reverse Transcriptase (Takara, Japan) in a total volume of 30 µl. Quantitative PCR was then carried out using the QuantStudio™ Design & Analysis System (ABI, USA) with the SYBR-Green Master mix (Yeasen, Shanghai, China). The relative RNA levels were normalized to *GAPDH* expression using the 2^−ΔΔCt^ method. The primer sequences used are listed in Supplementary Table 1.

### Western blot analysis

Protein was extracted from cultured cells by RIPA lysis buffer (Yeasen, Shanghai) with phenylmethylsulfonyl fluoride (PMSF) (1:100; Beyotime, China) and quantified with the BCA Protein Assay Kit (Beyotime). Protein lysates were separated on 15% sodium dodecyl sulfate-polyacrylamide gels and transferred to polyvinylidene difluoride membranes (0.22 μm; Millipore, Danvers, MA, USA). Then western blotting was carried out. In brief, the membranes were blocked with 5% milk at room temperature for 1 h, incubated with primary antibody overnight at 4 °C, and incubated with the appropriate horseradish peroxidase (HRP)-conjugated secondary antibodies (1:3000; Yeasen, Shanghai) for 1 h at room temperature. Proteins were detected by ECL reagent (Millipore). The results were obtained using a Tanon imager (Tanon, Shanghai, China). The primary antibodies used were as follows: PPARγ antibody (1:1000; Abcam), AP2 antibody (1:1000; Abcam), LPL antibody (1:1000; Abcam), CEBPα antibody (1:1000; Abcam), DSP antibody (25318-1-AP, 1:500; Proteintech, Wuhan, China), β-catenin antibody (51067-2-AP, 1:1000; Proteintech), GAPDH antibody (10494-1-AP, 1:10,000; Proteintech), and HRP-labeled ACTB antibody (1:10,000; Proteintech).

### siRNAs, ASOs, and lentivirus infection

siRNAs and ASOs used to knock down LYPLAL1-AS1 and the corresponding controls were purchased from Shanghai GeneBio (Shanghai, China), and siRNAs specific for DSP and the corresponding controls were purchased from RiboBio (Guangzhou, China). All siRNAs and ASOs were transfected into hAMSCs using Lipofectamine 2000 (Life Technology, USA) according to the manufacturer’s recommendations; the final siRNA or ASOs concentration used was 50 nM.

For overexpression, the full-length sequence of LYPLAL1-AS1 was inserted into the LV5-EF1-a-EGFP-Puro lentivirus expression vector and packaged by GenePharma (Shanghai, China), and a lentiviral vector that expressed scrambled RNA was used as the negative control. hAMSCs were infected with viral precipitates at a multiplicity of infection of 10, and stable cell lines were established by puromycin treatment

### Subcellular fractionation

The separation of nuclear and cytosolic fractions was performed using the NE-PER Nuclear and Cytoplasmic Extraction Reagents (Thermo Fisher Scientific, USA) according to the manufacturer’s instructions. RNA was extracted, and qRT-PCR was performed to assess the relative proportion in the nuclear and cytoplasmic fractions.

### FISH assay and colocalization of lncRNA with protein

FISH was performed using an RNA FISH Kit (RiboBio, Guangzhou, China). In brief, hAMSCs cultured on coverslips were rinsed in PBS and fixed with 4% formaldehyde for 10 min. Then, the cells were permeabilized in PBS containing 0.5% Triton X-100 at 4 °C for 5 min, washed three times, and prehybridized at 37 °C for 30 min. Then, anti-LYPLAL1-AS1, anti-U6, or anti-18S oligodeoxynucleotide probes (designed and made by RiboBio, Guangzhou, China) diluted in hybridization solution were incubated with the cells in the dark at 37 °C overnight. The next day, the cells were stained with 4′,6-diamidino-2-phenylindole (DAPI) and imaged using a fluorescence microscope (Carl Zeiss, Germany).

For immunofluorescence colocalization of lncRNA and protein, cells were fixed and permeabilized, and in situ hybridization with the LYPLAL1-AS1 oligodeoxynucleotide probe was carried out as described above. After that, the cell chamber was incubated with anti-DSP (1:50, 25318-1-AP; Proteintech) overnight at 4 °C. The next day, after being washed three times with PBS, the sample was incubated with Alexa Fluor 488-conjugated goat anti-rabbit secondary antibody (Thermo Fisher Scientific) at room temperature for 1 h, washed with PBS, and then incubated with DAPI for 5 min to dye the nuclei. The cells were imaged as described above.

### Rapid amplification of cDNA ends

RNA ligase-mediated RACE (RLM-RACE) was carried out with total RNA extracted from primary hAMSC cultures and was used to determine the transcription start site and the size of the LYPLAL1-AS1 transcript. 5′ and 3′ RACE was carried out using a FirstChoice RLM-RACE Kit (Thermo Fisher Scientific, Waltham, MA, USA). Because of the low copy number of LYPLAL1-AS1 transcripts in cells, nested PCR was performed for each reaction. The primers used for the nested PCR are listed in Supplementary Table 1.

### ChIRP-MS assay

hAMSCs were subjected to a ChIRP assay as described^58^. In brief, we carried out the following steps: (1) antisense RNA probes were designed to bind every 100 bp of the LYPLAL1-AS1 and U1 (positive control) transcripts, and the BiotinTEG biotin label was added to the 3′ end of each probe. (2) hAMSCs were cultured as described above, and 2 × 10^7^ hAMSCs induced for 3 days of adipogenic differentiation were collected. (3) For cell cross-linking and preservation, the cells were fixed with 1% glutaraldehyde for RNA–chromatin cross-linking. The samples were quickly frozen in liquid nitrogen and then stored in a −80 °C freezer. (4) Samples were added to lysis buffer supplemented with the protease inhibitor PMSF at room temperature. (5) Ultrasonic crushing was performed, and the cross-linked protein–RNA complexes were subjected to sonication. The lysate was checked every 30 min, and sonication continued until the cell lysate was no longer turbid. At this point, the DNA was broken into 100- to 500-bp fragments. (6) Hybridization of the cell lysate with biotinylated RNA probes (designed and synthesized by Aksomics, Shanghai, China) was then performed for ChIRP enrichment. (7) The extraction and subsequent analysis of RNA, DNA, and nucleic acid-binding proteins were carried out. The RNA isolated by ChIRP was extracted for qRT-PCR detection to confirm that the lncRNA molecule was a specific enrichment target, and GAPDH was used as a control. (8) Subsequent proteomics analyses were performed by Aksomics (Shanghai, China).

### RIP assay

A RIP assay was performed with the EZ-Magna RIP Kit (Millipore). Anti-DSP was purchased from Santa Cruz Biotechnology (Santa Cruz, CA, USA). RNAs that co-precipitated with DSP were extracted with TRIzol reagent, and LYPLAL1-AS1 enrichment was examined using qRT-PCR as we described above. Enrichment associated with normal rabbit IgG (Millipore) served as the control.

### De novo adipogenesis assay

This animal experiment was performed with approval by the Ethics Committee of the Chinese Academy of Medical Sciences and School of Basic Medicine Peking Union Medical College. We purchased 6-week-old NOD/SCID nude mice from SPF Biotechnology Co., Ltd (Beijing, China). Mice were housed under specific pathogen-free conditions for 2 weeks before the experiment was carried out. Three mice were randomly allocated to the LYPLAL1-AS1-overexpressing group and the control group. Specifically, 2 × 10^6^ hAMSCs induced for 3 days of adipogenic differentiation were suspended in 100 μL of adipogenic medium, mixed with 100 μL of Matrigel (BD Biosciences) on ice, and immediately injected subcutaneously into the dorsal surface of the mouse as we indicated in Fig S3a. Eight weeks later, each mouse was sacrificed and the de novo adipose mass was removed. Each adipose mass was fixed in 4% paraformaldehyde, embedded in paraffin, and sectioned (5 μm thick) for subsequent H&E staining and analysis.

### Immunohistochemical staining

Briefly, adipose mass was fixed in 4% PFA, dehydrated in 30% sucrose solution, and embedded in OCT. Samples were then sectioned (10-μm-thick sections) and incubated with GFP antibody (1:100, Servicebio, Wuhan, China) overnight at 4 °C, followed by incubation with HRP-conjugated secondary antibody (1:200, Servicebio, Wuhan, China) at room temperature for 1 h away from light. Cells were then visualized and imaged using a microscope.

### Statistical analysis

GraphPad Prism7 software (GraphPad Prism, San Diego, CA) was used for all statistical analyses, and data are expressed as the mean ± standard deviation. Student’s *t* test was used for statistical comparisons between the two groups. One-way analysis of variance was used for comparisons between multiple groups. Statistically significant differences were defined as follows: *P < 0.05, **P < 0.01, and ***P < 0.001.

## Supplementary information

Figure S1

Figure S2

Figure S3

Figure S4

Figure S5

Supplementary figure legends

Supplementary Table1

## References

[1] Tang QQ, Lane MD (2012). Adipogenesis: from stem cell to adipocyte. Annu. Rev. Biochem..

[2] KMaVJ. Dzau (2017). Mesenchymal stem cells in obesity: insights for translational applications. Lab. Investig..

[3] Lee RH (2004). Characterization and expression analysis of mesenchymal stem cells from human bone marrow and adipose tissue. Cell Physiol. Biochem..

[4] Payab M (2018). Stem cell and obesity: current state and future perspective. Adv. Exp. Med. Biol..

[5] Matsushita K, Dzau VJ (2017). Mesenchymal stem cells in obesity: insights for translational applications. Lab. Investig..

[6] Rinn JL, Chang HY (2012). Genome regulation by long noncoding RNAs. Annu. Rev. Biochem..

[7] Batista Pedro J, Chang Howard Y (2013). Long noncoding RNAs: cellular address codes in development and disease. Cell.

[8] Lee JT (2009). Lessons from X-chromosome inactivation: long ncRNA as guides and tethers to the epigenome. Genes Dev..

[9] Tsai MC (2010). Long noncoding RNA as modular scaffold of histone modification complexes. Science.

[10] Yang F (2011). Long noncoding RNA high expression in hepatocellular carcinoma facilitates tumor growth through enhancer of zeste homolog 2 in humans. Hepatology.

[11] Ulitsky I, Bartel DP (2013). lincRNAs: genomics, evolution, and mechanisms. Cell.

[12] Hu W, Alvarez-Dominguez JR, Lodish HF (2012). Regulation of mammalian cell differentiation by long non-coding RNAs. EMBO Rep..

[13] Peng WX, Koirala P, Mo YY (2017). LncRNA-mediated regulation of cell signaling in cancer. Oncogene.

[14] Sauvageau M (2013). Multiple knockout mouse models reveal lincRNAs are required for life and brain development. Elife.

[15] Gong C (2015). A long non-coding RNA, LncMyoD, regulates skeletal muscle differentiation by blocking IMP2-mediated mRNA translation. Dev. Cell.

[16] Grote P (2013). The tissue-specific lncRNA Fendrr is an essential regulator of heart and body wall development in the mouse. Dev. Cell.

[17] Li J (2019). Long noncoding RNA ANCR inhibits the differentiation of mesenchymal stem cells toward definitive endoderm by facilitating the association of PTBP1 with ID2. Cell Death Dis..

[18] Chen J, Wang Y, Wang C, Hu JF, Li W (2020). LncRNA functions as a new emerging epigenetic factor in determining the fate of stem cells. Front. Genet..

[19] Yuan H (2019). A novel long noncoding RNA PGC1β-OT1 regulates adipocyte and osteoblast differentiation through antagonizing miR-148a-3p. Cell Death Differ..

[20] Li M (2018). The long noncoding RNA GAS5 negatively regulates the adipogenic differentiation of MSCs by modulating the miR-18a/CTGF axis as a ceRNA. Cell Death Dis..

[21] Xiao T (2015). Long noncoding RNA ADINR regulates adipogenesis by transcriptionally activating C/EBPalpha. Stem Cell Rep..

[22] Liu C (2018). Long noncoding RNA H19 interacts with polypyrimidine tract-binding protein 1 to reprogram hepatic lipid homeostasis. Hepatology.

[23] Schmidt E (2018). LincRNA H19 protects from dietary obesity by constraining expression of monoallelic genes in brown fat. Nat. Commun..

[24] Nuermaimaiti N (2018). Effect of lncRNA HOXA11-AS1 on adipocyte differentiation in human adipose-derived stem cells. Biochem. Biophys. Res. Commun..

[25] Chen C (2018). Long non-coding RNAs regulation in adipogenesis and lipid metabolism: emerging insights in obesity. Cell Signal..

[26] Wang S (2019). Mesenchymal stem cells and immune disorders: from basic science to clinical transition. Front. Med..

[27] Li H (2018). MiRNA-10b reciprocally stimulates osteogenesis and inhibits adipogenesis partly through the TGF-β/SMAD2 signaling pathway. Aging Dis..

[28] White UA, Stephens JM (2010). Transcriptional factors that promote formation of white adipose tissue. Mol. Cell. Endocrinol..

[29] Suzuki Y (2012). The regulation of chemerin and CMKLR1 genes expression by TNF-α, adiponectin, and chemerin analog in bovine differentiated adipocytes. Asian-Australas. J. Anim. Sci..

[30] Spiegelman BM, Flier JS (1996). Adipogenesis and obesity: rounding out the big picture. Cell.

[31] Zhao Y (2014). p300-dependent acetylation of activating transcription factor 5 enhances C/EBPβ transactivation of C/EBPα during 3T3-L1 differentiation. Mol. Cell. Biol..

[32] Chu C (2015). Systematic discovery of Xist RNA binding proteins. Cell.

[33] Stubbins RE, Holcomb VB, Hong J, Núñez NP (2012). Estrogen modulates abdominal adiposity and protects female mice from obesity and impaired glucose tolerance. Eur. J. Nutr..

[34] Uldry M, Thorens B (2004). The SLC2 family of facilitated hexose and polyol transporters. Pflug. Arch..

[35] Fatima LA (2019). Estradiol stimulates adipogenesis and Slc2a4/GLUT4 expression via ESR1-mediated activation of CEBPA. Mol. Cell. Endocrinol..

[36] Lombardi R (2016). Cardiac fibro-adipocyte progenitors express desmosome proteins and preferentially differentiate to adipocytes upon deletion of the desmoplakin gene. Circ. Res..

[37] Feng YC (2020). c-Myc inactivation of p53 through the pan-cancer lncRNA MILIP drives cancer pathogenesis. Nat. Commun..

[38] Stavish D (2020). Generation and trapping of a mesoderm biased state of human pluripotency. Nat. Commun..

[39] Xu B (2010). Multiple roles for the non-coding RNA SRA in regulation of adipogenesis and insulin sensitivity. PLoS ONE.

[40] Gernapudi R (2016). MicroRNA 140 promotes expression of long noncoding RNA NEAT1 in adipogenesis. Mol. Cell. Biol..

[41] Zhang K (2014). The ways of action of long non-coding RNAs in cytoplasm and nucleus. Gene.

[42] Wilusz JE (2016). Long noncoding RNAs: re-writing dogmas of RNA processing and stability. Biochim. Biophys. Acta.

[43] da Rocha ST (2014). Jarid2 Is implicated in the initial Xist-induced targeting of PRC2 to the inactive X chromosome. Mol. Cell.

[44] Mellin JR (2013). A riboswitch-regulated antisense RNA in Listeria monocytogenes. Proc. Natl Acad. Sci. USA.

[45] Tripathi V (2010). The nuclear-retained noncoding RNA MALAT1 regulates alternative splicing by modulating SR splicing factor phosphorylation. Mol. Cell.

[46] Yoon JH, Abdelmohsen K, Gorospe M (2013). Posttranscriptional gene regulation by long noncoding RNA. J. Mol. Biol..

[47] Noh JH, Kim KM, McClusky WG, Abdelmohsen K, Gorospe M (2018). Cytoplasmic functions of long noncoding RNAs. Wiley Interdiscip. Rev. RNA.

[48] Delmar M, McKenna WJ (2010). The cardiac desmosome and arrhythmogenic cardiomyopathies: from gene to disease. Circ. Res..

[49] Dunn KE, Ashley EA (2015). Arrhythmogenic right ventricular cardiomyopathy: toward a modern clinical and genomic understanding. Circ. Cardiovasc. Genet..

[50] Yang F, Zhang H, Mei Y, Wu M (2014). Reciprocal regulation of HIF-1α and lincRNA-p21 modulates the Warburg effect. Mol. Cell.

[51] Mirza AH, Kaur S, Brorsson CA, Pociot F (2014). Effects of GWAS-associated genetic variants on lncRNAs within IBD and T1D candidate loci. PLoS ONE.

[52] Pan Y (2020). Long noncoding RNA repressor of adipogenesis negatively regulates the adipogenic differentiation of mesenchymal stem cells through the hnRNP A1-PTX3-ERK axis. Clin. Transl. Med..

[53] Cen S (2020). TRAF4 acts as a fate checkpoint to regulate the adipogenic differentiation of MSCs by activating PKM2. EBioMedicine.

[54] Li CH, Chen Y (2013). Targeting long non-coding RNAs in cancers: progress and prospects. Int. J. Biochem. Cell Biol..

[55] Cao Y (2005). Human adipose tissue-derived stem cells differentiate into endothelial cells in vitro and improve postnatal neovascularization in vivo. Biochem. Biophys. Res. Commun..

[56] Fan L (2018). miR-450b promotes osteogenic differentiation in vitro and enhances bone formation in vivo by targeting BMP3. Stem Cells Dev..

[57] Fan J (2018). MiR-1292 targets FZD4 to regulate senescence and osteogenic differentiation of stem cells in TE/SJ/mesenchymal tissue system via the Wnt/beta-catenin pathway. Aging Dis..

[58] Chu C, Quinn J, Chang HY (2012). Chromatin isolation by RNA purification (ChIRP). JoVE.

