# Supplementary figures and images for: Long noncoding RNA LYPLAL1-AS1 regulates adipogenic differentiation of human mesenchymal stem cells by targeting desmoplakin and inhibiting the Wnt/β-catenin pathway
Source: Cell Death Discov. 2021 May 15;7:105. doi: 10.1038/s41420-021-00500-5 (PMC8124068; doi:10.1038/s41420-021-00500-5)

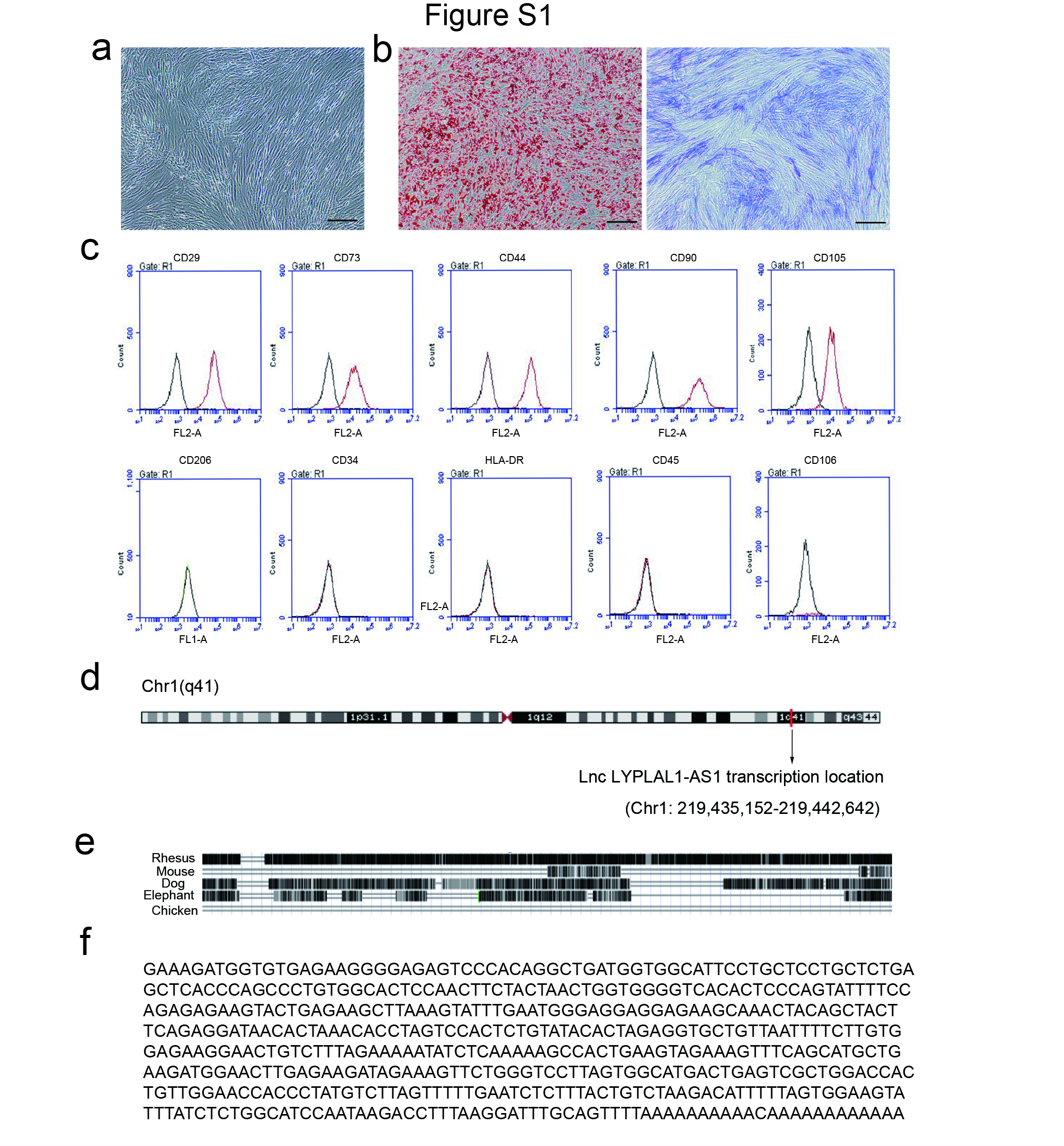

Supplement: Supplementary file 1 — Figure S1 [file 41420_2021_500_MOESM1_ESM.tif]

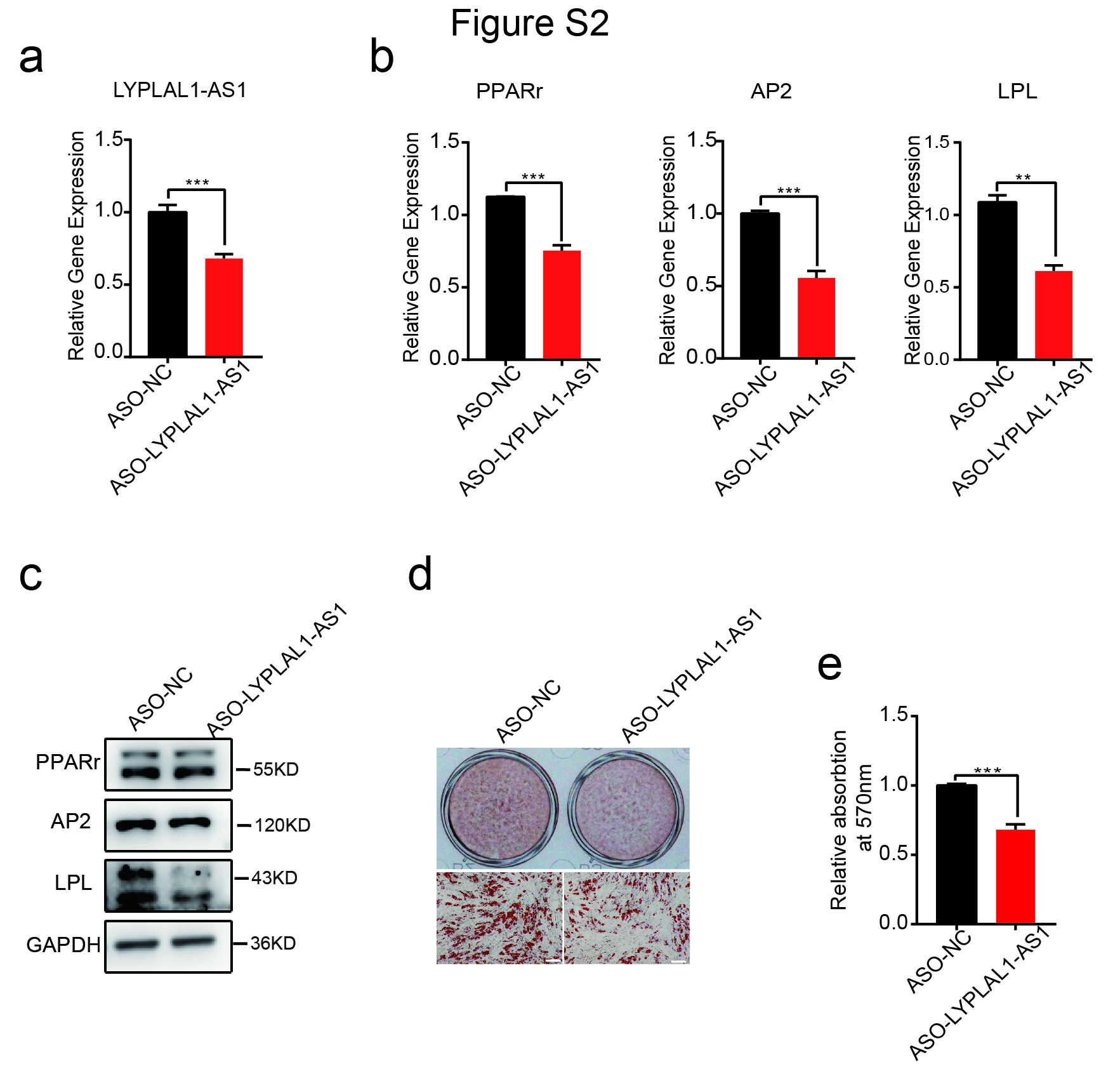

Supplement: Supplementary file 2 — Figure S2 [file 41420_2021_500_MOESM2_ESM.tif]

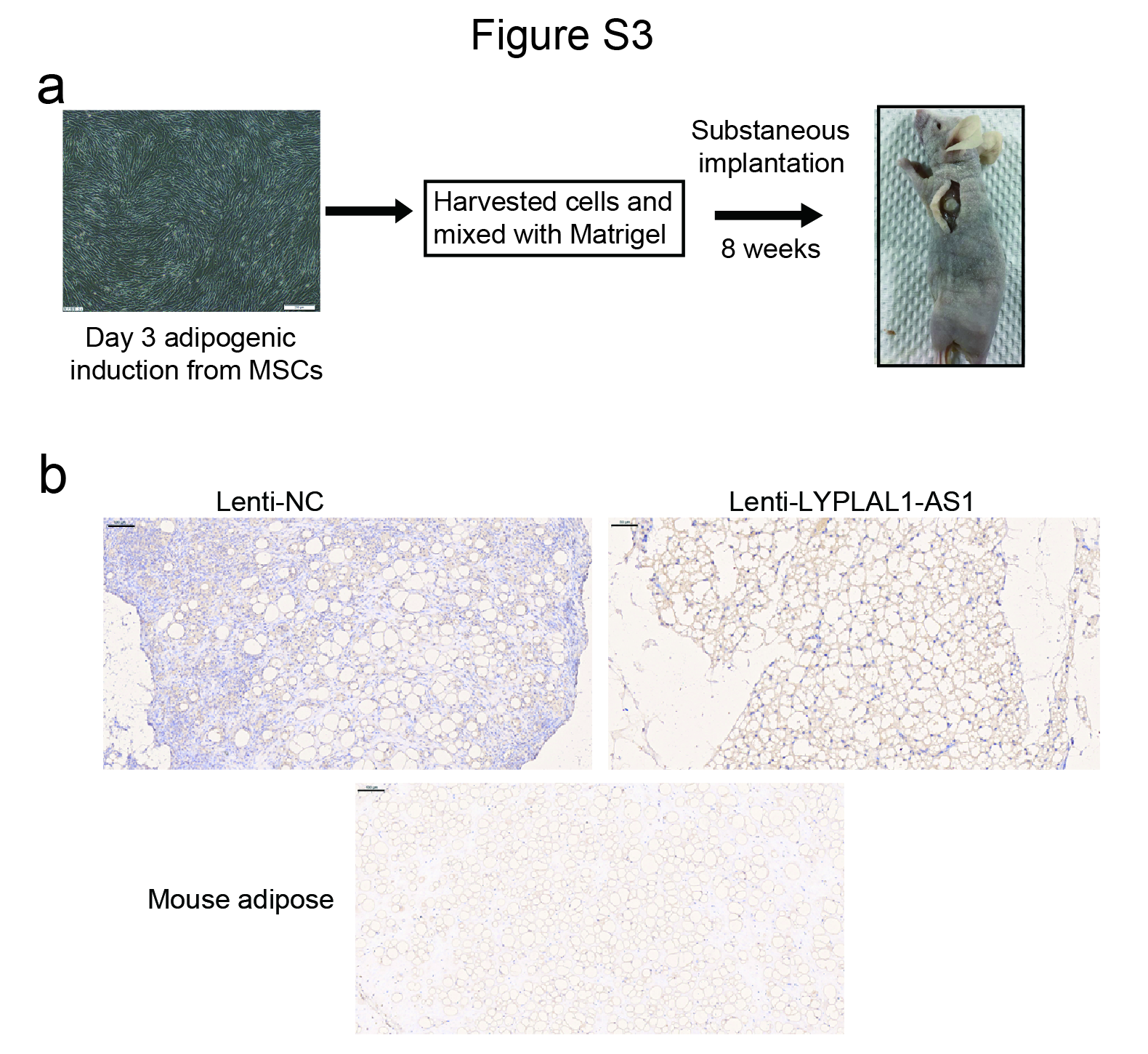

Supplement: Supplementary file 3 — Figure S3 [file 41420_2021_500_MOESM3_ESM.tif]

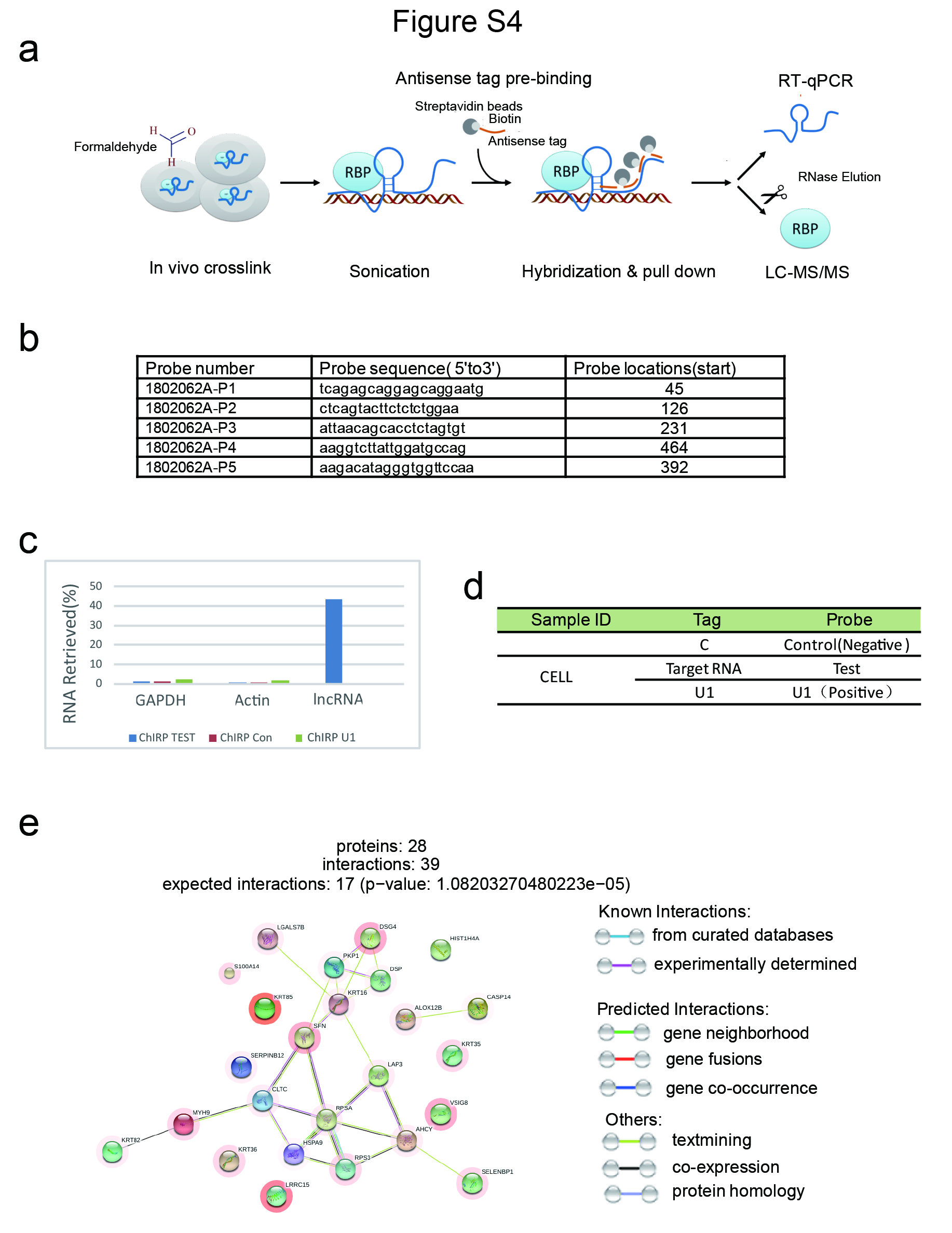

Supplement: Supplementary file 4 — Figure S4 [file 41420_2021_500_MOESM4_ESM.tif]

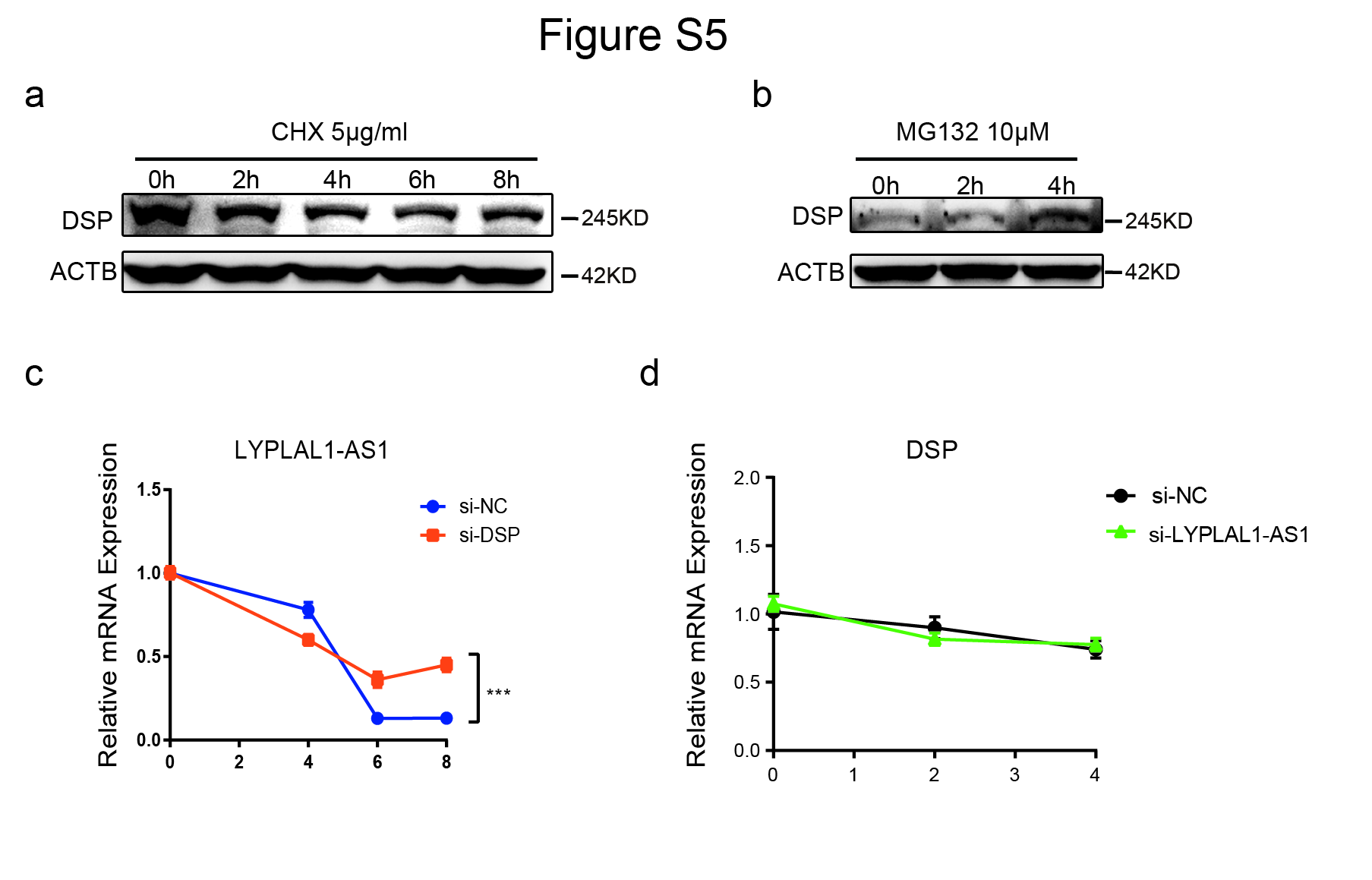

Supplement: Supplementary file 5 — Figure S5 [file 41420_2021_500_MOESM5_ESM.tif]
