# Supplementary material for: Long noncoding RNA LYPLAL1-AS1 regulates adipogenic differentiation of human mesenchymal stem cells by targeting desmoplakin and inhibiting the Wnt/β-catenin pathway
Source: Cell Death Discov. 2021 May 15;7:105. doi: 10.1038/s41420-021-00500-5 (PMC8124068; doi:10.1038/s41420-021-00500-5)
Supplement: Supplementary file 6 — Supplementary figure legends [file 41420_2021_500_MOESM6_ESM.docx]

**Supplementary figure legends**

**Figure S1.** **Morphological, functional, and phenotype identification of hAMSCs and** **characterization of LYPLAL1-AS1**

a. Morphology of hAMSCs under the light microscope.

b. Identification of differentiated hAMSC. hAMSCs were cultured for adipogenic differentiation for 10days and assayed for adipogenic differentiation with oil red O staining (left) or were cultured for osteogenic differentiation for 5days and assayed for osteogenic differentiation with ALP staining (right).

c. Flow cytometry identification of the hAMSC phenotype. Data are shown from a representative culture of hAMSCs at passage 3. Black curves represent isotype controls and red curves represent the specific antibodies.

d, e. Genome information of LYPLAL1-AS1 on the chromosome(d) and conservation analysis of LYPLAL1-AS1(e) according to the UCSC Genome Browser.

f. The full sequence of LYPLAL1-AS1 shown from the 5’ to 3’ ends was confirmed by 5’ RACE and 3’ RACE.

**Figure S2. Knockdown of LYPLAL1-AS1 using ASO inhibits the adipogenic differentiation of hAMSCs**

a. LYPLAL1-AS1 was silenced in hAMSCs using RNase-H−based antisense oligonucleotides (ASO-LYPLAL1-AS1). The knockdown efficiency was verified by qRT-PCR as compared with cells treated with the negative control (ASO-NC).

b, c. qRT-PCR analysis (b) and western blot analysis (c) of adipogenic differentiation markers (PPARγ, AP2, and LPL) in ASO-LYPLAL1-AS1−treated hAMSCs and control hAMSCs on day 3 after adipogenic induction. GAPDH was used as the internal control for western blotting.

d, e. Oil red O staining of adipose lipids in ASO-LYPLAL1-AS1−treated hAMSCs and control hAMSCs on day 10 after adipogenic induction (d) and quantification of the oil red O staining (e).

GAPDH was used as the internal control for the western blot assays. Quantitative data were normalized to GAPDH, n = 3; data are shown as the mean ± S.D.; **P < 0.01, ***P < 0.001; scale bars: 200 µm.

**Figure S3. *De novo* adipogenesis procedure and exogenous GFP immunohistochemical staining related to Figure 3.**

a. Schematic of the *in vivo* experimental setup. hAMSCs were infected with lentivirus (Lenti-NC, Lenti-LYPLAL1-AS1) and induced to undergo adipogenic differentiation for 3 days. Cells were then harvested and mixed with Matrigel before being subcutaneously injected into NOD/SCID nude mice. The adipose plugs were collected 8 weeks later for analysis.

b. Immunohistochemical staining for exogenous GFP in adipose plugs from mice injected with Lenti-LYPLAL1-AS1 and Lenti-NC hAMSCs (upper). Mouse shoulder fat (adipose) was used as a negative control (lower). scale bars: 100 µm.

**Figure S4. ChIRP-MS−identified proteins that bind LYPLAL1-AS1 (related to Figure 4)**

a. Technical procedure for ChIRP-MS.

b. Five specific probes labeled with biotin were used to pull down the proteins that directly bind to LYPLAL1-AS1.

c. Over 40% of LYPLAL1-AS1 was selectively pulled down without enrichment of the housekeeping control GAPDH or the control U1 snRNA.

d. Quantification group and sample information.

e. Protein interaction analysis of the specific binding proteins using STRINGdb revealed significant enrichment in interactions among the 28 identified proteins.

**Figure S5. Protein stability and degradation of DSP, and mRNA decay of DSP and LYPLAL1-AS1**

a. DSP protein stability detected by western blotting. hAMSCs were treated with cycloheximide (CHX, 5 μg/ml) for 0, 2, 4, 6, and 8 h. Cells were then harvested and subjected to western blotting for DSP and ACTB (control).

b. hAMSCs were treated with MG132 (10 μM) for 0, 2, and 4 h. Cells were then harvested and subjected to western blotting for DSP and ACTB (control).

c, d. mRNA decay detected by qRT-PCR in DSP knockdown (c) and LYPLAL1-AS1 knockdown (d) hAMSCs. hAMSCs transfected with si-NC or si-DSP (c) and si-NC or si- LYPLAL1-AS1 (d) were treated with 5 μg/ml actinomycin D. At different time points, total RNA was extracted, and LYPLAL1-AS1 mRNA was analyzed by qRT-PCR and normalized to GAPDH. mRNA at 0 h served as a reference.

Data are shown as the mean ± SD (n = 3). ***P < 0.001.
