# Supplementary material for: Long noncoding RNA LYPLAL1-AS1 regulates adipogenic differentiation of human mesenchymal stem cells by targeting desmoplakin and inhibiting the Wnt/β-catenin pathway
Source: Cell Death Discov. 2021 May 15;7:105. doi: 10.1038/s41420-021-00500-5 (PMC8124068; doi:10.1038/s41420-021-00500-5)
Supplement: Supplementary file 7 — Supplementary Table1 [file 41420_2021_500_MOESM7_ESM.docx]

**Supplementary Table 1. The sequence of primers used in this study.**

| **Gene** | **Primer sequence** (5’-3’) |
| --- | --- |
| LYPLAL1-AS1 | F: GAGGAGGAGAAGCAAACTACAG |
|  | R: GACTCAGTCATGCCACTAAGG |
| Desmoplakin  (DSP) | F: GGAAGTGCATGGGAGGATAAG |
|  | R: ATGTTGTAAGGGCTGGGTAAG |
| PPAR-γ | F: TGAACGTGAAGCCCATCGAG |
|  | R: CTTGGCGAACAGCTGAGAGG |
| AP2 | F: ACTGGGCCAGGAATTTGACG |
|  | R: CTCGTGGAAGTGACGCCTT |
| LPL | F: TCATTCCCGGAGTAGCAGAGT |
|  | R: GGCCACAAGTTTTGGCACC |
| CEBPα | F: GCGCAAGAGCCGAGATAAAG |
|  | R: CGGTCATTGTCACTGGTCAACT |
| β-catenin | F: GCCAAGTGGGTGGTATAGAG |
|  | R: CTGGGTATCCTGATGTGC |
| GAPDH | F: GGTCACCAGGGCTGCTTTTA |
|  | R: GGATCTCGCTCCTGGAAGATG |
| 5' RACE Outer | F: GCTGATGGCGATGAATGAACACTG |
|  | R: GGTCTTATTGGATGCCAGAGAT |
| 3' RACE Outer1 | F: GGCTGTTTCATCCTGGGTATT |
|  | R: GCGAGCACAGAATTAATACGACT |
| 5' RACE Inner | F: CGCGGATCCGAACACTGCGTTTGCTGGCTTTGATG |
|  | GSP-R: AGTGGACTAGGTGTTTAGTGTTATC |
| 3' RACE Inner | GSP-F: GAGGAGGAGAAGCAAACTACAG |
|  | R: CGCGGATCCGAATTAATACGACTCACTATAGG |
